# The association of CYP2D6 gene polymorphisms in the full-length coding region with higher recurrence rate of vivax malaria in Yunnan Province, China

**DOI:** 10.1186/s12936-021-03685-3

**Published:** 2021-03-20

**Authors:** Herong Huang, Ying Dong, Yanchun Xu, Yan Deng, Canglin Zhang, Shuping Liu, Mengni Chen, Yan Liu

**Affiliations:** 1 School of Basic Medical Sciences, Dali University, Dali, 667000 China; 2 Yunnan Institute of Parasitic Diseases Control, Yunnan Provincial Key Laboratory of Vector-Borne Diseases Control and Research, Yunnan Centre of Malaria Research, Pu’er, 665000 China

**Keywords:** *Plasmodium vivax*, Relapse, CYP2D6 gene, Coding region, Primaquine, Allele, Genotype

## Abstract

**Background:**

Accumulating evidence suggest that compromised CYP2D6 enzyme activity caused by gene mutation could contribute to primaquine failure for the radical cure of vivax malaria. The current study aims to preliminarily reveal the association between the recurrence of vivax malaria in Yunnan Province and CYP2D6 gene mutation by analysing polymorphisms in the entire coding region of human CYP2D6 gene.

**Methods:**

Blood samples were collected from patients with vivax malaria, who received "chloroquine and 8-day course of primaquine therapy" in Yunnan Province. The suspected relapsed cases were determined by epidemiological approaches and gene sequence alignment. PCR was conducted to amplify the CYP2D6 gene in the human genome, and the amplified products were then sequenced to compare with the non-mutation “reference” sequence, so as to ensure correct sequencing results and to determine 9 exon regions. Subsequently, the DNA sequences of 9 exons were spliced into the coding DNA sequence (CDS), which, by default, is known as maternal CDS. The paternal CDS was obtained by adjusting the bases according to the sequencing peaks. The mutation loci, haplotypes (star alleles), genotypes and odds ratios (OR) of all the CDSs were analysed.

**Results:**

Of the119 maternal CDS chains in total with 1491 bp in length, 12 mutation sites in the 238 maternal and paternal CDS chains were detected. The c.408G > C mutation was most frequently detected in both suspected relapsed group (SR) and non-relapsed group (NR), reaching 85.2% (75/88) and 76.0% (114/150), respectively. The c.886C > T mutation was most closely related to the recurrence of vivax malaria (OR = 2.167, 95% CI 1.104–4.252, P < 0.05). Among the 23 haplotypes (Hap_1 ~ Hap_23), Hap_3 was non-mutant, and the sequence structure of Hap_9 was the most complicated one. Five star alleles, including *1, *2, *4, *10 and *39, were confirmed by comparison, and CYP2D6*10 allele accounted for the largest percentage (45.4%, 108/238). The frequency of CYP2D6*2 allele in the SR group was significantly higher than that in the NR group (*Χ*^*2*^ = 16.177, P < 0.05). Of the defined 24 genotypes, 8 genotypes, including *4/*4, *4/*o, *2/*39, *39/*m, *39/*x, *1/*r, *1/*n, and *v/*10, were detected only in the SR group.

**Conclusion:**

Mutation of CYP2D6*10 allele accounts for the highest proportion of vivax malaria cases in Yunnan Province. The mutations of c. 886C > T and CYP2D6*2 allele, which correspond to impaired PQ metabolizer phenotype, are most closely related to the relapse of vivax malaria. In addition, the genotype *4/*4 with null CYP2D6 enzyme function was only detected in the SR group. These results reveal the risk of defected CYP2D6 enzyme activity that diminishes the therapeutic effect of primaquine on vivax malaria.

**Supplementary Information:**

The online version contains supplementary material available at 10.1186/s12936-021-03685-3.

## Background

In recent years, the global malaria epidemic has gradually scaled down, yet the death tolls remain high and the number of malaria cases in certain areas has increased [1]. Meanwhile, multiple technological restrains, including the difficulty to identify low-density *Plasmodium* infection [2], the expansion of drug resistant *Plasmodium* [3, 4], and the lack of new drugs in the anti-malarial pipeline [5], pose daunting challenges to the elimination of malaria. Although the local cases of malaria have not been reported since 2016 [6], China still needs to cope with a large number of imported malaria cases, especially from Africa and Southeast Asian countries [7]. From 2011 to 2018, 50.7% of the imported malaria cases in Yunnan Province were caused by *Plasmodium vivax* [8]. Therefore, the elimination of malaria calls for the strict enforcement to block the transmission of *P. vivax*.

Tafenoquine and primaquine are recommended by the World Health Organization (WHO) for the treatment of relapsed malaria owing to its efficiency of eradicating the hypnozoites of *P. vivax* via 5-hydroxy-primaquine (5-hydroxy-primaquine) formed by the enzyme CYP2D6 (Cytochrome P450, family 2, subfamily D, polypeptide 6) in human liver cells [9–12]. CYP2D6, also known as debrisoquine4-hydroxylase, is an isozyme in the Cytochrome P450 (CYP450) super family and an important phase I drug-metabolizing enzyme. Owing to its low capacity and high affinity, CYP2D6 is involved in the metabolism of ~ 30% of commonly used drugs, including primaquine although it only accounts for 4% of the total P450 enzyme protein in the liver [12]. Studies have found that the heterogeneity of CYP2D6 activity is predominantly governed by its genetic variation [13], and that decreased CYP2D6 isoenzyme activity caused by genetic polymorphisms would obstruct the generation of 5-hydroxy-primaquine, making large doses or repeated use of primaquine necessary to compensate for the declined efficacy of primaquine. Meanwhile, primaquine can cause life threatening haemolysis in patients with glucose-6-phosphate dehydrogenase (G6PD) deficiency, subsequently inflicting low efficacy in anti-relapse and acute hemolysis [9, 14, 15]. Therefore, the risk of G6PD deficiency should be identified prior to receiving anti-relapse treatment of vivax malaria, and that the genotype and enzyme activity of CYP2D6 should be tested as well [14, 16, 17].

CYP2D6 enzyme activity is measured mainly through phenotypic observation and genotypic analysis. Compared with phenotypic characterization, genotypic prediction manifests the advantages of greater stability, more convenience and being less affected by environmental or physiological factors [18–20]. CYP2D6 enzyme activity can be detected from the blood sample of the patients; genotypic sequencing is currently the common technical method to determine the allelic form of CYP2D6 gene [21–25] by identifying the locus status of the CYP2D6 gene [13] or allelic combinations [26, 27], thereby predicting the activity level of CYP2D6. However, as the number of alleles of CYP2D6 gene continues to increase, efforts should be made to improve the precision and inclusiveness of genotyping results [28]. The better understanding of the genetic polymorphism of CYP2D6, which metabolizes about 25% of all medications in the human liver, can not only help deal with individual adverse event and but also enable the more precise DNA genotyping-based prediction.

Primaquine is commonly used in the malaria endemic areas of Yunnan Province, yet the systematic survey on the genetic polymorphisms of CYP2D6 has been not conducted. In this study, Whole Genome Sequencing (WGS) was conducted to investigate the polymorphisms of CYP2D6 gene in the vivax malaria cases, thereby revealing the association between human CYP2D6 gene polymorphisms and the relapsed cases of vivax malaria.

## Methods

### Study subjects and sampling

A total of 120 subjects living in Yunnan province, China (97°31′ E to 106°11′ E; 21°8′ N to 29°15′ N) were enrolled in the study. The subjects were diagnosed of mono-infection of *P. vivax* by both microscopy examination and *Plasmodium* 18S rRNA gene detection (PCR) in Yunnan Province Malaria Diagnosis Referent Laboratory (YPMDRL) (Additional file 1). All the patients received oral chloroquine therapy (1550 mg in total) within 3 days, followed by the subsequent 8-day course of primaquine therapy (22.5 mg/day).

The subjects diagnosed from 2014 to 2018 were subdivided into suspected relapse of vivax malaria (SR group) and non-relapse of vivax malaria (NR group). The inclusion criteria of the SR group are: (1) the patients were re-diagnosed as *P. vivax* infection within the interval of 28–180 days after the initial diagnosis; (2) epidemiological investigation confirmed that the patients were not re-exposed to malaria endemic areas between the two malaria episodes; and (3) the comparative sequencing of CSP (Circumsporozoite Surface Protein) and MSP-1 (Merozoite Surface Protein 1) of *P. vivax* with those of the previously infected strains found 100% similarity and 0 variable sites.

A simple random sampling method was conducted to selected 75 NR cases from the 143 patients confirmed in 2018. The subjects in the NR group had no history of relapsed vivax malaria after 1 year of follow-up. The source of vivax malaria infection was determined through epidemiological investigations. The subjects had no travelling history to malaria endemic areas within 30 days before the onset of malaria were regarded as local cases, otherwise they were regarded as imported cases. The study was approved by Yunnan Institute of Parasitic Diseases and Ethical Committee document No. 201904 (January 25, 2019). Genotyping was performed by using the stored blood samples obtained from the suspected patients who experienced fever and chills. Informed consent was obtained from all the patients. The blood samples of the subjects were provided by the Centers for Disease Control and Prevention in Yunnan Province, according to the described protocol [29]. Dried blood spots (DBS) sampling was conducted by collected 0.6 ml of venous blood, which was stored in a dried tube at − 80 ℃ before the extraction of DNA.

### Extraction of genomic DNA

Three dried blood spots (diameter = 5 mm) were made, and genomic DNA was extracted according to the manufacturer’s instructions of the QIAgen Mini Kit (Germany, QIAamp Company's DNA Mini Kit), according to the manufacture’s instruction. The extracted DNA was stored at − 20 ℃.

### PCR amplification of CYP2D6 gene fragment

The primers of CYP2D6 for polymerase chain reaction (PCR) amplification were designed by using GenBank (https://www.ncbi.nlm.nih.gov/gene/), reference sequence (ID: NC_000022.11) was used as template, following the reaction condition in previous studies [23, 30]. The primers for first-round nested PCR covering the coding region of CYP2D6 gene exons1–4 were: 5′-CCAGTGACAGATAAGGGTGC-3′ and 5′-GACGTGGATAGGAGGTAC AGAG-3′; the primers for second-round nested PCR were: 5′-GGTGACTTCTCCGACCAG G-3′ and 5′-TTCCCAAACCCATCTATGC-3′. The final amplification region was 42,131,088–42,128,678, and the expected fragment length of the product was 2411 bp. The primers that cover exons 5–9 of CYP2D6 gene were: 5′-GCCGACTGAGCCCTGGGA GGTAGGTA-3′ and 5′-GCTGGGGCCTGAGACTT-3. The amplification area was 42,126,035–42,128,422, and the amplification products had an expected fragment length of 2388 bp. For all the PCR reaction systems, we used 2.6 μl DNA template, 14.0 μl 2 × Taq PCR hybrid system (QIAGEN, Germany), 0.7 μl upstream primer (20umol/L), 0.7 μl downstream primers (20umol/L). The total volume was adjusted to 25.0 μl with ddH_2_O. PCR reaction conditions were clarified as follows: 92 to 94 °C for 2–5 min; 92 to 94 °C for 10–30 s, 50 to 56 °C for 15–30 s, 68 to 72 °C for 2 to 3 min, 35 cycles; 68 to 72 °C for 7 min. The triplicated parallel repetition was adopted for each PCR reaction. The amplified products were observed using 1.5% agarose gel electrophoresis. The positive amplification products were sent to Shanghai Meiji Biomedical Technology Co, Ltd. for sequencing by using the Sanger method. Only the sequences that showed identical results in at least two tests were used for subsequent analysis.

### CYP2D6 gene mutations analysis

The sequencing reads were aligned by using DNAStar and BioEdit software packages. The obtained DNA sequences were assessed by using the Basic Local Alignment Search Tool (BLAST, http://blast.ncbi.nlm.nih.gov/Blast.cgi) on the NCBI platform. The sequences having a homology of 100% and query cover of above 99% with the non-mutated “reference” sequence (ID: NC_000022.11) were considered as the human CYP2D6 gene sequence.

The sequence was further compared with the non-mutation reference sequence to identify 9 exon regions, the transcriptional starting point and the end point of CYP2D6 gene. The DNA sequences of the 9 exon regions were spliced into the maternal CDS (coding DNA sequence) of CYP2D6 gene in the order from exon 1 to exon 9. The paternal CDS was obtained by replacing the undisplayed diploid bases on the maternal CDS chain [31, 32], and the diploid bases were determined by searching the sequencing peaks (Additional file 2). This approach makes up for the shortcoming that the sequencer can only read one base signal for each site, whereas misses the other hybrid base. MEGA v5.04 software was used to analyse the missense mutations and synonymous mutation sites of maternal and paternal CDS, and the ID number of the defined mutation site was queried in Genbank. DnaSP 6.11.01 software was used to identify the haplotype of the CDS, to calculate the nucleic acid diversity index (π), expected heterozygosity (He) and other parameters [33].

The haplotype (allelic form) of CDSs was determined according to the criteria of Human Cytochrome P450 Allele Nomenclature Committee (NM_000106.6) [34] and previous study [35], and were termed to the level of sub-alleles (such as *10.001 and *39.001). The sub-alleles were then merged. Those haplotypes unmatched with known CYP2D6 alleles were grouped into the “other” category. The genotype of each sample was a diploid composed of the maternal CDS and parental CDS (such as *10/*39 and *1/*10). SPSS software (version 21.0; IBN; Chicago; IL) was used to conduct chi-square test on the frequency of genotypes and mutation loci co-existed in the SR cases and NR cases. The odds ratio (OR) of genotypes and mutation loci with statistical significance were calculated by comparing to the relapse rate of *P. vivax*. The significance level was set as *P* < 0.05; OR < 1 indicates that the haplotype is a protective factor, while OR < 1 indicates a risk factor.

## Results

### Demographics and clinical characteristics of the subjects and PCR amplification of human CYP2D6 gene

A total of 45 SR vivax malaria cases were confirmed by epidemiological investigation and gene sequence alignment (Additional file 3), including 43 cases with one suspected relapsed event, 1 case with two suspected relapsed events and 1 case with three suspected relapsed events. The demographic and clinical characteristics of the subjects are listed in Table 1. The male–female ratio was 3:1. Most of the patients presented only one suspected relapsed episode (95.6%).

**Table 1:** Demographic and clinical characteristics of the study cohort

Variable	Total (n, F%)	SR group (n, F%)	NR group (n, F%)
Total	120 (100.0)	45 (37.5)	75 (62.5)
Gender			
Male	90 (75.0)	32 (71.1)	51 (68.0)
Female	30 (25.0)	13 (28.9)	24 (32.0)
Age (in years)			
0–20	22 (18.3)	5 (11.1)	17 (22.7)
21–60	89 (74.2)	38 (84.5)	51 (68.0)
above 60	9 (7.5)	2 (4.4)	7 (9.3)
Malaria relapse			
1 episode	43 (35.8)	43 (95.6)	–
2 episodes	1 (0.8)	1 (2.2)	–
3 episodes	1 (0.8)	1 (2.2)	–
Infection source^a^			
Myanmar	117 (97.5)	42 (93.4)	75 (100.0)
Africa	1 (0.8)	1 (2.2)	–
Yunnan indigenous	2 (1.7)	2 (4.4)	–

*n* number of cases, *F* Frequency

^a^Identified by epidemiological investigation

Exons 1–4 and 5–9 of CYP2D6 gene in the blood samples collected from 45 SR patients and 75 NR patients were amplified by conducting PCR. The amplified product revealed a clear band at > 2000 bp, and therefore can be considered as the target band (Additional file 4). The CYP2D6 gene fragments of 75 NR patients and 44 SR patients were successfully amplified, expect for the PCR product of CYP2D6 gene was not effectively amplified for 1 SR patient.

### Locus polymorphisms of CDS and its association with relapsed cases of vivax malaria

A total of 119 maternal CDS of CYP2D6 gene (Genbank accession number: MT339075-MT339193) were obtained. Base substitutions at 12 loci, including c.31 and c.100, on the 238 maternal and paternal CDS, were determined (Table 2). The proportions of third-base and first-base substitution in the triplet codon were 41.7% (5/12), and the proportion of second-base substitution was 16.6% (2/12). 7 missense mutation loci and 5 synonymous mutation loci were determined. 91.7% (11/12) of the mutation sites were found in the sequences of SR group, while 66.7% (8/12) of the mutation sites were determined in the sequences of NS group (Table 2).

**Table 2:** Polymorphisms of CDS mutation in CYP2D6 gene between different vivax malaria cases

SR group				NR group					OR^c^	95%CI		*P*	SNP ID in Genbank
Loci	Codon change^a^	Amino acid change	No (n = 88, %^b1^)	Loci	Codon change^a^	Amino acid change	No. (n = 150, %^b2^)			Upper limit	Low limit		
c.31	GTG > ***A***TG	V11M	1 (0.7)	–	–	–		–	–	–	–	–	rs769258
c.100	CCA > ***T***CA	P34S	47 (53.4)	c.100	CCA > ***T***CA	P34S		95 (63.3)	0.664	0.389	1.133	0.132^d^ (NS)	rs1065852
c.271	CTG > ***A***TG	L91M	4 (4.5)	c.271	CTG > ***T***TG	L91L		2 (1.3)	3.524	0.632	19.647	0.272^d^ (NS)	rs28371703
c.281	CAC > C***G***C	H94R	4 (4.5)	–	–	–		–	–	–	–	–	rs28371704
c.294	ACC > AC***G***	T98T	4 (4.5)	c.294	ACC > AC***G***	T98T		1 (0.7)	7.095	0.780	64.521	0.122^d^ (NS)	rs28371705
c.297	GCC > GC***T***	A99A	1 (1.4)	–	–	–		–	–	–	–	–	rs200269944
c.336	TTC > TT***T***	F112F	42 (47.7)	c.336	TTC > TT***T***	F112F		81 (54.0)	0.778	0.459	1.318	0.350^d^ (NS)	rs1081003
c.408	GTG > GT***C***	V136V	75 (85.2)	c.408	GTG > GT***C***	V136V		114 (76.0)	1.822	0.907	3.661	0.089^d^ (NS)	rs1058164
c.505	GGT > ***A***GT	G169S	2 (2.3)	–	–	–		–	–	–	–	–	rs5030865
–	–	–	–	c.801	CCC > CC***A***	P267P		2 (1.3)	–	–	–	–	rs28371718
c.886	CGC > ***T***GC	R296C	22 (23.9)	c.886	CGC > ***T***GC	R296C		20 (13.3)	2.167	1.104	4.252	0.023^d^ (S)	rs16947
c.1457	AGC > A***C***C	S486T	75 (85.2)	c.1457	AGC > A***C***C	S486T		108 (72.0)	1.416	0.819	2.447	0.212^d^ (NS)	rs1135840

The comparative results of 12 single nucleotide polymorphisms (SNPs) between the two groups were listed in Table 2. In the SR group, the frequency of mutation allele was the highest at c.1457 G > C (85.2%, 75/88), and c. 408 G > C (85.2%,75/88). In the NR group, the highest frequency of mutation was at c. 408 G > C (76.0%, 114/150), followed by c. 1457 G > C (72.0%, 108/150). Eight mutation loci found both in SR group and NR group were selected to analyse the association between loci mutation and vivax malaria relapse. Correlation analysis showed that the ratio of c.886C > T site mutation to the occurrence of vivax malaria recurrence was 2.167 (95% CI 1.104–4.252), and that statistically significance was determined (P˂ 0.05). Such result suggests that c.886 mutation increases the risk of recurrence of vivax malaria, which is 2.167 times higher than that of other SNPs (Table 2).

### Haplotype diversity

**Fig. 1:**
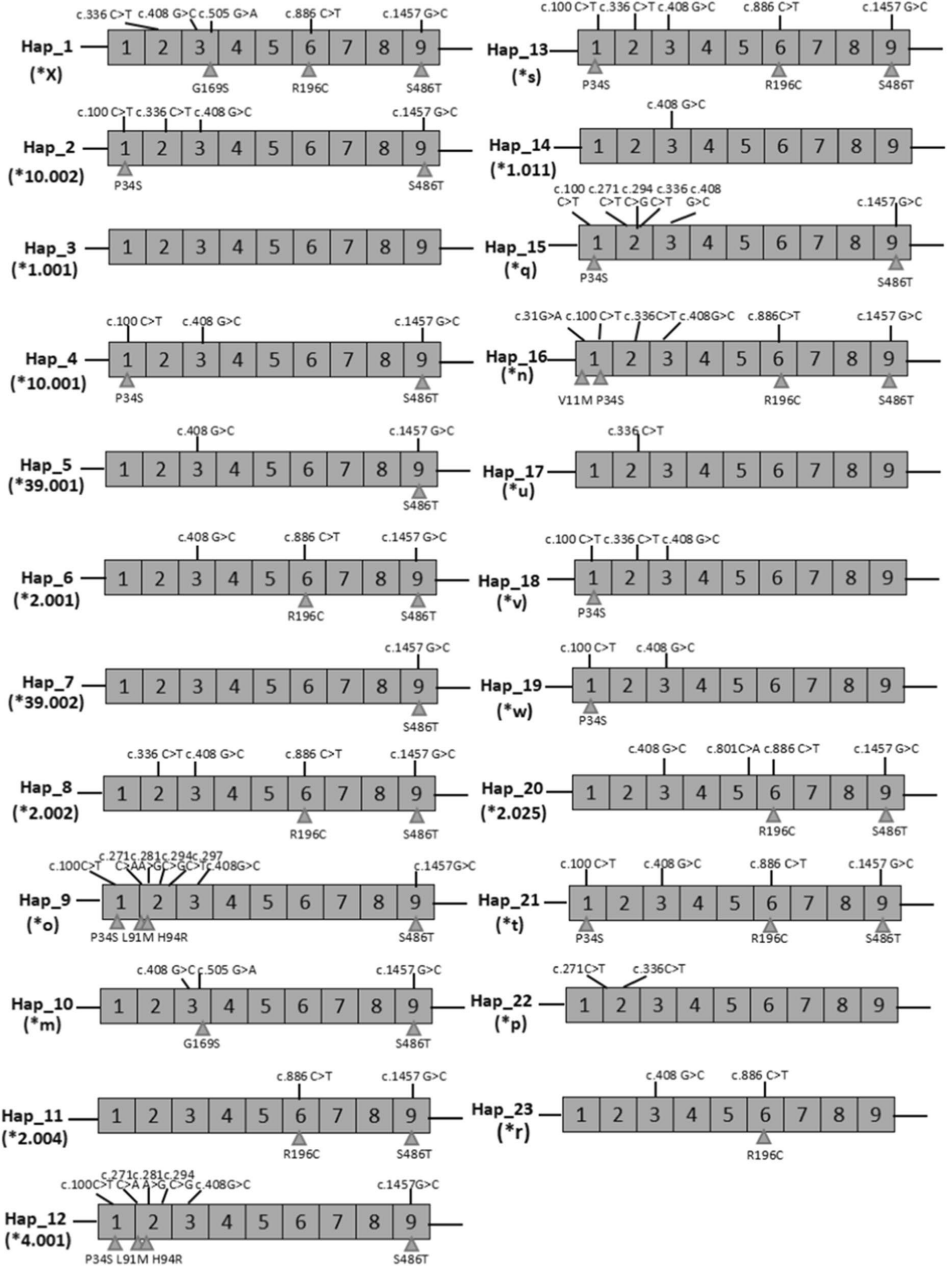
Haplotype structure of CYP2D6 gene. 9 exons are indicated by numbered boxes with DNA polymorphisms indicated on top. Predicted amino acid changes are indicated below; no change is synonymous mutation. Hap: Haplotypes; *m-*x: They could not meet the allele inclusion criteria provided by the Allele Nomenclature Committee

A total of 23 haplotypes (Hap_1 ~ Hap_23) were identified amongst the 238 CDSs of CYP2D6 gene. Of them, 9 haplotypes were only observed in the sequences of SR group (π = 0.0015, He = 0.821), while 6 haplotypes were only found in the NR group (π = 0.0014 and He = 0.760). 8 haplotypes, including Hap_2, Hap_3, Hap_4, Hap_5, Hap_6, Hap_7, Hap_13 and Hap_14, were found in both SR and NR group. The compositions of every haplotype were shown in Fig. 1. Hap_3 did not contain mutation loci, and the rest haplotypes contained different mutation loci.

### Alleles/genotypes and their association with vivax malaria relapse

Among the 23 haplotypes, 11 sub-alleles (such as *1.001, *1.011, *2.001 and *2.004) that could be accurately termed belonged to 5 star (*) alleles, including *1, *2, *4, *10 and *39. Moreover, 12 sub-alleles (*m-*x) had no referencing information. The most common alleles included *10 (45.4%, 108/238), *1 (19.7%, 47/238) and *39 (11.8%, 28/238) (Table 3). In addition, *10 alleles accounted for the highest proportion in both SR group and NR group, reaching the frequencies of 38.6% (34/88) and 49.3% (74/150), respectively, yet the difference did not reach statistically significant level (*x*^*2*^ = 2.560, *P* > 0.05). The frequency of alleles *1 in the NR group (*x*^*2*^ = 6.193, *P* < 0.05), and the frequency of alleles *2 in SR group was significantly higher (*x*^*2*^ = 16.177, *P* < 0.05).

**Table 3:** The association between CYP2D6 genotypes and the relapse of vivax malaria

Alleles	Total No (n1 = 238, F/%)	Case groups		*x*^*2*^	*P*
		SR group No. (n1 = 88, F/%)	NR group No. (n1 = 150, F/%)		
a. Alleles					
*1	47 (19.7)	10 (11.4)	37 (24.7)	6.193	0.013^b^ (S)
*2	17 (7.1)	14 (15.9)	3 (2.0)	16.177	0.000^b^ (S)
*4	3 (1.3)	3 (3.4)	0	2.802	0.094^b^ (NS)
*10	108 (45.4)	34 (38.6)	74 (49.3)	2.560	0.110^b^ (NS)
*39	28 (11.8)	15 (17.0)	13 (8.7)	3.751	0.053^b^ (NS)
*s^a^	20 (8.4)	5 (5.7)	15 (10.0)	1.344	0.246^b^ (NS)
Other^a^	15 (6.3)	7 (8.0)	8 (5.3)	1.766	0.184^b^ (NS)

Genotypes	Case groups		OR^c^	95%CI		*P*
	SR group No. (n2 = 44, F/%)	NR group No. (n2 = 75, F/%)		Upper limit	Low limit	
b. Genotypes						
*1/*1	1 (2.3)	11 (14.7)	0.135	0.017	1.087	0.064^b^ (NS)
*2/*2	4 (9.1)	1 (1.3)	7.400	0.800	68.463	0.118^b^ (NS)
*1/*2	4 (9.1)	1 (1.3)	7.400	0.800	68.463	0.118^b^ (NS)
*1/*10	2 (4.5)	12 (16.0)	0.250	0.050	1.174	0.115^b^ (NS)
*10/*10	12 (27.3)	24 (32.0)	0.797	0.350	1.873	0.588^b^ (NS)
*10/*s^a^	1 (2.3)	1 (1.3)	1.721	0.105	28.220	1.000^b^ (NS)
*10/*39	5 (11.4)	3 (4.0)	3.077	0.698	13.563	0.242^b^ (NS)
*39/*39	2 (4.5)	1 (1.3)	3.524	0.310	40.032	0.636^b^ (NS)
*39/*s^a^	2 (4.5)	7 (9.3)	0.463	0.092	2.332	0.552^b^ (NS)

*n1* number of chromosomes, *n2* number of cases, *F* Frequency

Other: *m, *n, *o, *p, *q,*r,*t, *u, *v, *w and *x; ^a^ They could not meet the allele inclusion criteria provided by the Allele Nomenclature Committee [32]; ^b^Chi-square test; NS: not significant (*P* > 0.05); ^c^The OR value of genotypes were calculated by comparing to the relapse rate of *P. vivax*; S: significant (*P* < 0.05); NS: not significant (*P* > 0.05)

The current study defined 32 sub-allelic genotypes, which can be classified into 24 star alleles genotypes (Additional file 5). Among them, the frequency of *10/*10 was the highest in both SR group and NR group, reaching 24.2% (12/44) and 32.0% (24/75), respectively (Additional file 5). In addition, 8 sub-allelic genotypes, including *4/*4, *4/*o, *2/*39, *39/*m, *39/*x, *1/*r, *1/*n, *v/*10, were only detected in SR group. We conducted correlational analysis on the 9 genotypes distributed in both two groups (including *1/*1, *2/*2, *1/*2, *1/*10, *10/*10, *10/ *39, *39/*39, *10/*s and *39/*s), and revealed that the odd ratios of all genotypes were not statistically significant (P > 0.05) (Table 3). This result suggested that the different CYP2D6 genotypes in this study did not affect the recurrence of vivax malaria.

## Discussion

Located on chromosome 22q13.1, CYP2D6 gene (4383 bp in length) consists of 8–9 exons and 7–8 introns. Its CDS is 1338–1491 bp in length and encodes 446–497 amino acids. The heterogeneity of CYP2D6 monooxygenase activity varies from complete dysfunction to ultra-rapid enzyme metabolism, depending on the various genotypes of CYP2D6 [14]. Among the 150 identified alleles, multiple mutations such as CYP2D6*3, CYP2D6*4, CYP2D6*5 and CYP2D6*6 are considered to represent null CYP2D6 enzyme activity [30, 32, 33, 36].

The current study is the first one that attempts to analyse CYP2D6 polymorphisms in the entire coding region sequence in the blood specimen of relapsed vivax malaria patients in Yunnan. The current study reveals the association between CYP2D6 polymorphisms and thwarted CYP2D6 enzyme activity, which is accountable to the failed treatment of using primaquine. This study found that mutation at c. 886 locus of CYP2D6 gene elevated the risk of vivax malaria relapse risk by 2.167-fold (*P* < 0.05). In the findings of Wang et al*.* [37] and Gaedigk et al*.* [38], it is concluded that the relapse of vivax malaria may be relate to c.886. locus mutation of CYP2D6 gene in Yunnan Province.

Among the 23 alleles defined by the 23 haplotypes in all the CDSs, CYP2D6*10 accounts for the highest proportion, reaching as high as 45.4% (108/238). Consistently, previous studies found the highest frequency of CYP2D6*10 alleles mutation in the Chinese Han population and in Asian subjects [31, 39–41]. Although the frequency of CYP2D6*10 alleles did not show statistical significant difference between the SR group and the SR group, CYP2D6*10 mutation was considered to indicate decreased enzyme activity [24, 34]. This notion suggested that the wide distribution of CYP2D6 gene*10 alleles in the population of Yunnan should not be disregarded, as it will amplify the accumulated risk of reduced therapeutic efficacy of primaquine in eliminating *P. vivax* hypnozoites in Yunnan Province.

The current study found that CYP2D*2 allele was more frequently determined in the relapsed patients. This result was not in agreement with that of Brasil et al*.* [30], which reported that the frequency of CYP2D*2 mutation was significantly higher in the non-relapsed patients. Such inconsistent results might be attributed to the different subjects enrolled in the two studies. Specifically, the subjects included in this study were vivax malaria patients who live in the malaria endemic areas in Yunnan Province, hence the long-term malaria epidemic environment could positively screen the human defective genes [42–44].

In this study, all the alleles were identified based on the mutations in the exon regions of CYP2D6, which is different from allelic recognition that covers intron mutations [32, 45]. Therefore, the homogeneity of CYP2D6*2 allele in the two studies could not be ruled out. Although CYP2D6*2 allele is generally considered to be an allelic type of CYP2D6 normal enzyme activity, multiple mutation that contains c. 886 locus could result in lowered activity of CYP2D6 enzyme [38, 42]. At present, the complicated multi-directional change of CYP2D6 enzyme activity caused by *2 allelic mutation is yet to be validated [35, 37, 38, 45].

Eight mutant allelic types were found only in the SR group, yet the association between these different CYP2D6 genotypes and the relapse of vivax malaria could not be established. Given that the *10/*10 genotype still accounted for the largest proportion (30.3%) in the overall study cohort and the no-function *4/*4 genotype was found in the SR group [39], the risk of declined therapeutic efficacy of primaquine caused by defective CYP2D6 enzyme activity in malaria endemic areas should not be ignored. In addition, effort should be made to validate whether the one case of amplification failure case in this study was caused by full gene deletion (*5/*5) or other factors.

The limitations of the current study should be noted. Firstly, the sample size of suspected recurrence of vivax malaria is not large enough, and the alleles (*4, *m-*x,) of CYP2D6 genes with low frequency of distribution might not be quantitatively analyzed (Additional file 5). Secondly, while the re-infection with *P. vivax* was ruled out from the SR group, the influences of primaquine doses and primaquine resistance were not evaluated. Finally, polymorphisms in the entire coding region of CYP2D6 gene were investigated yetthe possible mutation in intron region and the copy number of CYP2D6 gene, which has been considered to disturb the activity of CYP2D6 enzyme, were not analysed. Thusly, the activity of CYP2D6 cannot be indirectly predicted based on the results of CYP2D6 genotyping in the present study. In view of such shortcoming, the sampling size should be expanded to conduct molecular epidemiological investigations on CYP2D6 genotype.

Currently, the number of CYP2D6 gene CDS has increased to 320, and consistent results pertaining to the loci mutation and allelic type were found. Moreover, multiplex ligation-dependent probe amplification (MLPA) and other advanced methods will be applied to optimize the accurate identification of human CYP2D6 genotypes, so as to swiftly predict CYP2D6 enzyme activity of individual subjects. These findings will contribute to the efficacy of primaquine in the treatment of vivax malaria in Yunnan province.

## Conclusions

This pilot study reveals the polymorphisms of CYP2D6 gene coding region and its association with the recurrence of vivax malaria in the population of Yunnan Province, suggesting that the radical cure of vivax malaria by using primaquine could be hampered due to the impaired CYP2D6 enzyme activity in the population of Yunnan Province. The study revealed that (1) CYP2D6*10 allele accounts for the highest proportion in the vivax malaria cases of Yunnan Province; (2) mutation at c. 886 locus is most closely related to the relapse of vivax malaria and CYP2D6*2 allele containing this mutation locus was significantly higher in SR; and (3) The *4/*4 genotype that indicates null CYP2D6 enzyme function (poor metabolizers) was only found in the SR cases.

## Supplementary Information

**Additional file 1.** The details of nested PCR testing for differentiating between various *Plasmodium* species.

**Additional file 2.** Sequencing Peak Map of CYP2D6 Gene Polymorphic loci.

**Additional file 3.** Epidemiological information and alignment results of the genes of suspected recurrent cases of vivax malaria.

**Additional file 4.** Electrophoretic map of PCR products in the coding region of CYP2D6 gene.

**Additional file 5.** Analysis the CYP2D6 genotypes in SR group and NR group.

## Data Availability

Not applicable.

## Abbreviations

WHO: World Health Organization
CYP2D6: Cytochrome P450, family 2, subfamily D, polypeptide 6
G6PD: Glucose-6-Phosphate Dehydrogenase
CDS: Coding DNA sequence
PCR: Polymerase chain reaction
YPMDRL: Yunnan Province Malaria Diagnosis Referent Laboratory
CDC: Center for Disease Control
DBS: Dried blood spots
NR: Non-relapsed cases of vivax malaria
SR: Suspected relapsed cases of vivax malaria
*csp*: *circumsporozoite surface protein*
*msp-1*: *merozoite surface protein 1*
Hap: Haplotype
π: Diversity index
He: Expected heterozygosity
OR: Odds ratio
CI: Confidence interval
MPLA: Multiplex ligation-dependent probe amplification
SNP: Single nucleotide polymorphism
