# Supplementary material for: The association of CYP2D6 gene polymorphisms in the full-length coding region with higher recurrence rate of vivax malaria in Yunnan Province, China
Source: Malar J. 2021 Mar 20;20:160. doi: 10.1186/s12936-021-03685-3 (PMC7981985; doi:10.1186/s12936-021-03685-3)

**Additional file 2**

Sequencing peak map of polymorphic loci

1. c.31 G>A

Mutant heterozygote: There is an overlap of A and G base signals at 298 of the sequencing peak map (as shown in the box in the figure).


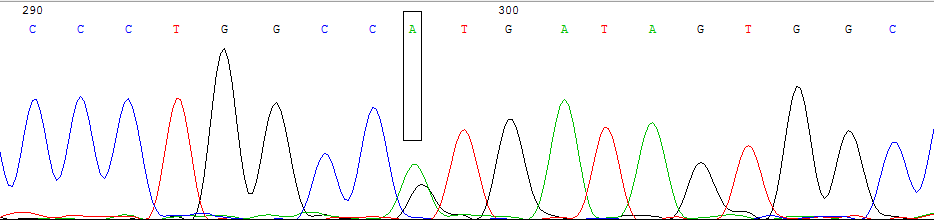


1. c.100 C>T

2.1 Mutant homozygous: Base C is replaced by T at 369 of the sequencing peak map (as shown in the box in the figure).


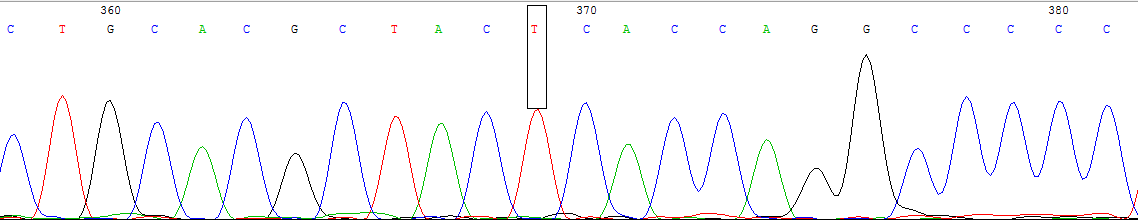


2.2 Mutant heterozygote: There is an overlap of C and T base signals at 365 of the sequencing peak map (as shown in the box in the figure).


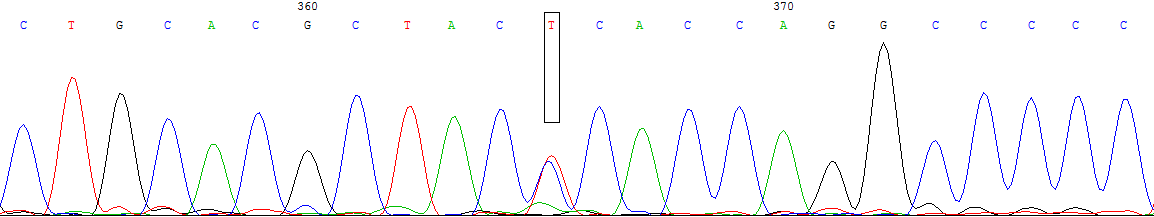


1. c.271 C>A

3.1 Mutant homozygous: Base C is replaced by A at 378 of the sequencing peak map (as shown in the box in the figure).


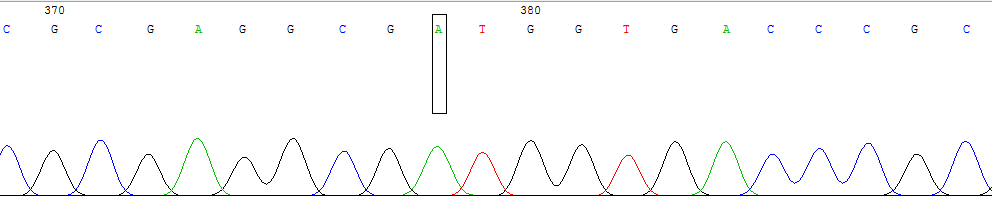


3.2 Mutant homozygous: Base C is replaced by T at 373 of the sequencing peak map (as shown in the box in the figure).


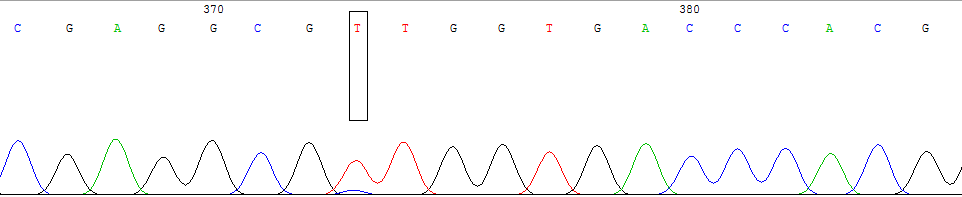


1. c.281 A>G

Mutant homozygous: Base A is replaced by G at 381 of the sequencing peak map (as shown in the box in the figure).


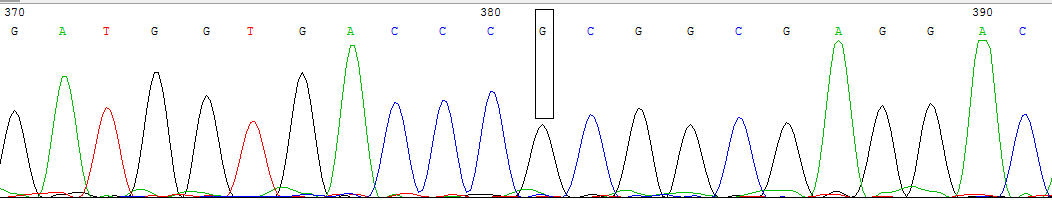


1. c.294 C>G

Mutant homozygous: Base C is replaced by G at 394 of the sequencing peak map (as shown in the box in the figure).


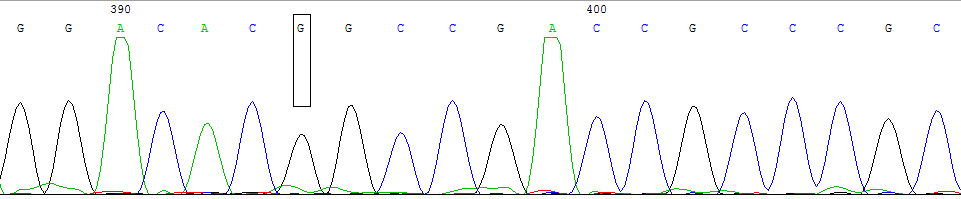


1. c.297 C>T

Mutant heterozygote: There is an overlap of C and T base signals at 404 of the sequencing peak map (as shown in the box in the figure).


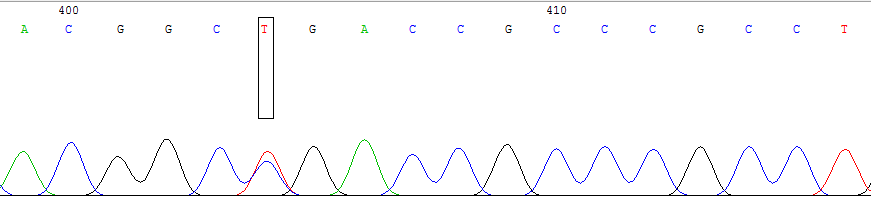


1. c.336 C>T

7.1 Mutant homozygous: Base C is replaced by T at 436 of the sequencing peak map (as shown in the box in the figure).


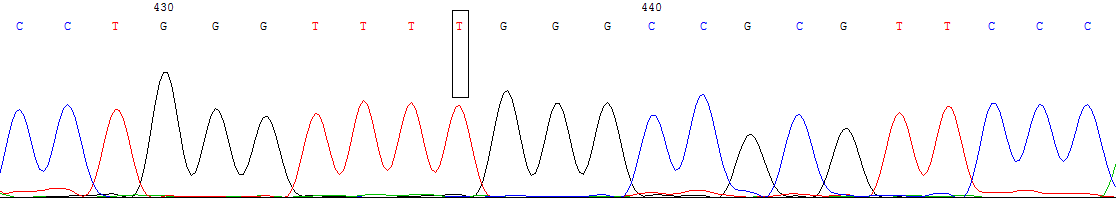


7.2 Mutant heterozygote: There is an overlap of C and T base signals at 437 of the sequencing peak map (as shown in the box in the figure).


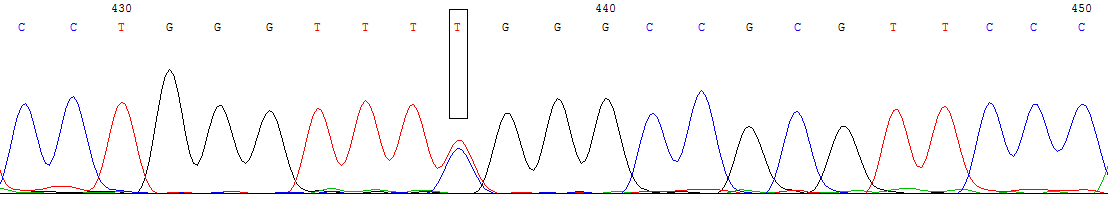


1. c.408 G>C

8.1 Mutant homozygous: Base G is replaced by C at 436 of the sequencing peak map (as shown in the box in the figure).


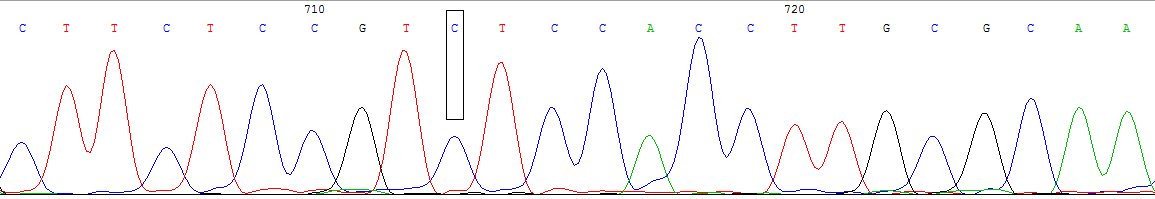


8.2 Mutant heterozygote: There is an overlap of G and C base signals at 499 of the sequencing peak map (as shown in the box in the figure).


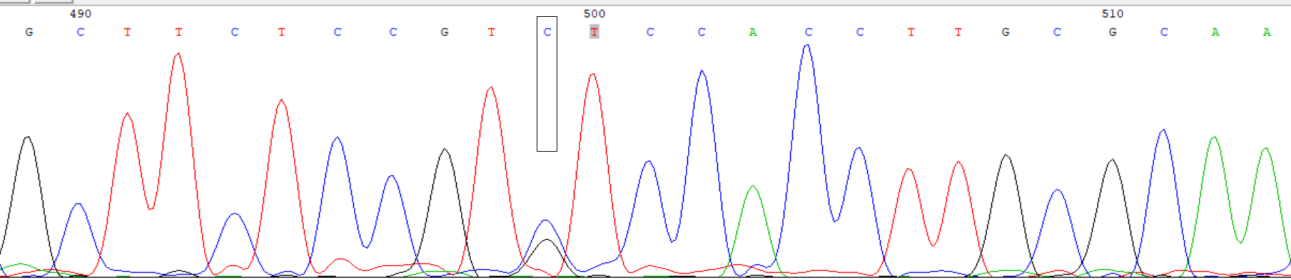


1. c.505 G>A

Mutant heterozygote: There is an overlap of G and A base signals at 553 of the sequencing peak map (as shown in the box in the figure).


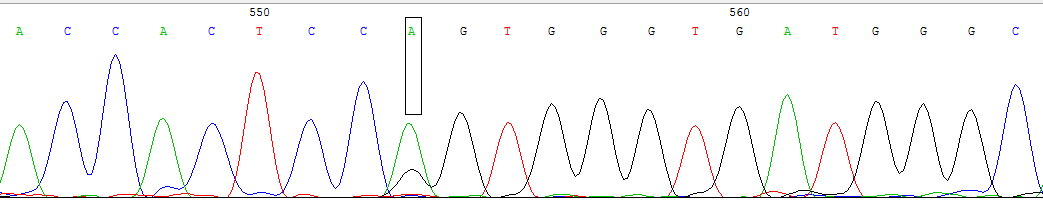


1. c.801 C>A

Mutant homozygous: Base C is replaced by A at 178 of the sequencing peak map (as shown in the box in the figure).


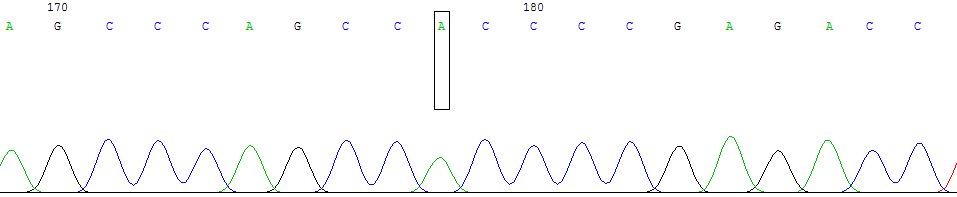


1. c.886 C>T

11.1 Mutant homozygous: Base C is replaced by T at 451 of the sequencing peak map (as shown in the box in the figure).


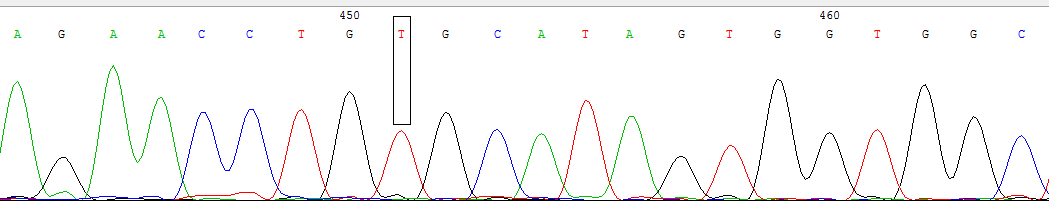


11.2 Mutant heterozygote: There is an overlap of C and T base signals at 455 of the sequencing peak map (as shown in the box in the figure).


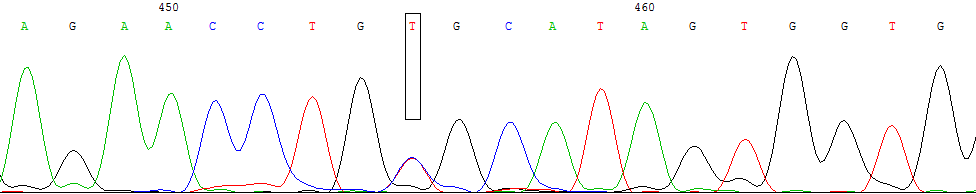


1. c.1457 G>C

12.1 Mutant homozygous: Base G is replaced by C at position 599 of the sequencing peak map (as shown in the box in the figure).


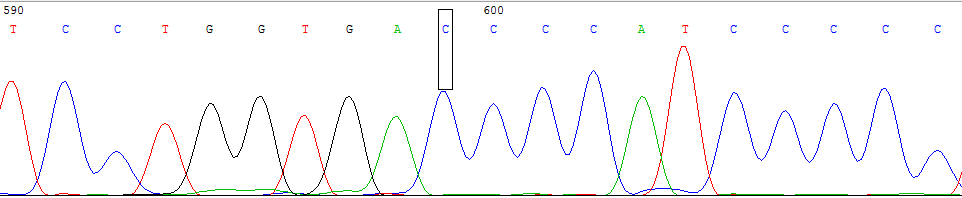


12.2 Mutant heterozygote: There is an overlap of G and C base signals at 514 of the sequencing peak map (as shown in the box in the figure).


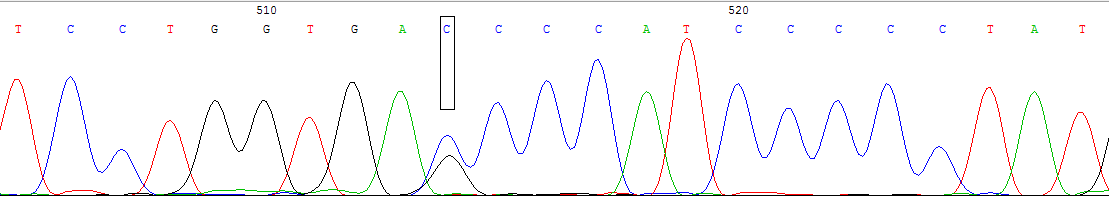

Supplement: Supplementary file 2 — Additional file 2. Sequencing Peak Map of CYP2D6 Gene Polymorphic loci. [file 12936_2021_3685_MOESM2_ESM.docx]
