# Supplementary material for: The association of CYP2D6 gene polymorphisms in the full-length coding region with higher recurrence rate of vivax malaria in Yunnan Province, China
Source: Malar J. 2021 Mar 20;20:160. doi: 10.1186/s12936-021-03685-3 (PMC7981985; doi:10.1186/s12936-021-03685-3)

| **Additional file 3 Epidemiological information and alignment results of the genes of suspected recurrent cases of vivax malaria** | | | | | |
| --- | --- | --- | --- | --- | --- |
| Cases | Interval of recurrence (in days) | Epidemiological investigation^a^ | Genetic comparisons^d^ | | Characteristics of cases |
|  |  |  | *pvcsp* | *pvmsp-1* |  |
| Case 1 | 176 | No re-exposed | Variable sites=0 | Variable sites=0 | suspected recurrence |
| Case 2 | 171 | No re-exposed | Variable sites=0 | Variable sites=0 | suspected recurrence |
| Case 3 | 168 | No re-exposed | Variable sites=0 | Variable sites=0 | suspected recurrence |
| Case 4 | 178 | No re-exposed | Variable sites=0 | Variable sites=0 | suspected recurrence |
| Case 5 | 177 | No re-exposed | Variable sites=0 | Variable sites=0 | suspected recurrence |
| Case 6 | 85 | No re-exposed | Variable sites=0 | Variable sites=0 | suspected recurrence |
| Case 7 | 175 | No re-exposed | Variable sites=0 | Variable sites=0 | suspected recurrence |
| Case 8 | 58 | No re-exposed | Variable sites=0 | Variable sites=0 | suspected recurrence |
| Case 9 | 178 | No re-exposed | Variable sites=0 | Variable sites=0 | suspected recurrence |
| Case 10 | 62 | No re-exposed | Variable sites=0 | Variable sites=0 | suspected recurrence |
| Case 11 | 60 | No re-exposed | Variable sites=0 | Variable sites=0 | suspected recurrence |
| Case 12 | 179 | No re-exposed | Variable sites=0 | Variable sites=0 | suspected recurrence |
| Case 13 | 162 | No re-exposed | Variable sites=0 | Variable sites=0 | suspected recurrence |
| Case 14 | 160 | No re-exposed | Variable sites=0 | Variable sites=0 | suspected recurrence |
| Case 15 | 130 | No re-exposed | Variable sites=0 | Variable sites=0 | suspected recurrence |
| Case 16^b^ | 165/122/97 | No re-exposed | Variable sites=0 | Variable sites=0 | suspected recurrence |
| Case 17 | 169 | No re-exposed | Variable sites=0 | Variable sites=0 | suspected recurrence |
| Case 18 | 171 | No re-exposed | Variable sites=0 | Variable sites=0 | suspected recurrence |
| Case 19 | 175 | No re-exposed | Variable sites=0 | Variable sites=0 | suspected recurrence |
| Case 20 | 71 | No re-exposed | Variable sites=0 | Variable sites=0 | suspected recurrence |
| Case 21 | 54 | No re-exposed | Variable sites=0 | Variable sites=0 | suspected recurrence |
| Case 22 | 45 | No re-exposed | Variable sites=0 | Variable sites=0 | suspected recurrence |
| Case 23 | 178 | No re-exposed | -- | Variable sites=0 | suspected recurrence |
| Case 24 | 166 | No re-exposed | Variable sites=0 | Variable sites=0 | suspected recurrence |
| Case 25 | 177 | No re-exposed | -- | Variable sites=0 | suspected recurrence |
| Case 26 | 162 | No re-exposed | Variable sites=0 | -- | suspected recurrence |
| Case 27 | 45 | No re-exposed | Variable sites=0 | Variable sites=0 | suspected recurrence |
| Case 28 | 88 | No re-exposed | Variable sites=0 | Variable sites=0 | suspected recurrence |
| Case 29 | 174 | No re-exposed | -- | Variable sites=0 | suspected recurrence |
| Case 30 | 180 | No re-exposed | -- | Variable sites=0 | suspected recurrence |
| Case 31 | 128 | No re-exposed | -- | Variable sites=0 | suspected recurrence |
| Case 32 | 172 | No re-exposed | -- | Variable sites=0 | suspected recurrence |
| Case 33 | 90 | No re-exposed | Variable sites=0 | -- | suspected recurrence |
| Case 34 | 173 | No re-exposed | Variable sites=0 | Variable sites=0 | suspected recurrence |
| Case 35 | 167 | No re-exposed | -- | Variable sites=0 | suspected recurrence |
| Case 36 | 174 | No re-exposed | -- | Variable sites=0 | suspected recurrence |
| Case 37 | 97 | No re-exposed | Variable sites=0 | Variable sites=0 | suspected recurrence |
| Case 38 | 180 | No re-exposed | Variable sites=0 | Variable sites=0 | suspected recurrence |
| Case 39 | 177 | No re-exposed | Variable sites=0 | Variable sites=0 | suspected recurrence |
| Case 40 | 162 | No re-exposed | Variable sites=0 | Variable sites=0 | suspected recurrence |
| Case 41 | 178 | No re-exposed | Variable sites=0 | Variable sites=0 | suspected recurrence |
| Case 42^c^ | 68/66 | No re-exposed | Variable sites=0 | Variable sites=0 | suspected recurrence |
| Case 43 | 178 | No re-exposed | -- | Variable sites=0 | suspected recurrence |
| Case 44 | 177 | No re-exposed | -- | Variable sites=0 | suspected recurrence |
| Case 45 | 176 | No re-exposed | Variable sites=0 | Variable sites=0 | suspected recurrence |
| Note: ^a^Through epidemiological investigation, whether they were re-exposed to malaria transmission area between the two clinical malaria attacks. The epidemiological investigation is carried out jointly by the local disease control and research group.  ^b^ Three relapses of vivax malaria. ^c^ Two relapses of vivax malaria. ^c^ The results of gene sequence alignment of every vivax malaria recurrent cases is shown later. | | | | | |

Identification of the isolates between vivax malaria initial and suspected recurrent cases based on the polymorphism of *pvcsp* gene & *pvmsp-1* gene fragment

**Method：**

The primers of *pvcsp* gene & *pvmsp-1* gene for polymerase chain reaction (PCR) amplification were designed by using GenBank (https://www.ncbi.nlm.nih.gov/gene/), reference sequence (ID：LT635619.1 and LT635618.1) were used as template. The details of primers and amplification region of every round PCR showed in Table 1. For all the PCR reaction systems, we used 2.6μl DNA template, 14.0μl 2 × Taq PCR hybrid system (QIAGEN, Germany), 0.7μl upstream primer (20umol / L), 0.7μl downstream primers (20umol / L), and the total volume for PCR reaction was adjusted to 25.0μl with ddH2O. The positive amplification products were sent to Shanghai Meiji Biomedical Technology Co, Ltd. for sequencing by Sanger dideoxy sequencing.

The sequencing results were aligned by using DNAStar 5.10 and BioEdit 7.0.9.0 software. The obtained all DNA sequences were assessed by using the Basic Local Alignment Search Tool (BLAST, http://blast.ncbi.nlm.nih.gov/Blast.cgi) in the NCBI platform. Every DNA sequence with identifications was bigger than 99% and Query cover above 96% were considered as objective gene sequence of *P. vivax*. The DnaSP 6.11.01 software was used to identify haplotypes of DNA sequences and calculate the Variable sites and Expected heterozygosity (He).

| **Table 1 The details of nested PCR for amplification the genes of *P. vivax*** | | | | | |
| --- | --- | --- | --- | --- | --- |
| **Gene** | **Nested PCR** | **Sequence of primers** | **Prodect length (bp)** | **Amplification region** | **Referent sequence** |
| *pvcsp* | First round | 5-ATGTAGATCTGTCCAAGGCCATAAA-3' | 1018 | 1508787-1509804 | LT635619.1  (Chromosome 8) |
|  |  | 5'-TAATTGAATAATGCTAGGACTAACAATATG-3' |  |  |  |
|  | Second round | 5'-GCAGAACCAAAAAATCCACGTGAAAATAAG-3' | 641 | 1508957-1509597 |  |
|  |  | 5'-CCAACGGTAGCTCTAACTTTATCTAGGTAT-3' |  |  |  |
|  |  |  |  |  |  |
| *pvmsp-1* | First round | 5'-CCCTACTACTTGATGGTCCTCA-3' | 733 | 1217406-1218138 | LT635618.1  (Chromosome 7) |
|  |  | 5'-CCTTCTGGTACAACTCAATG-3' |  |  |  |
|  | Second round | 5'-AGCATGATCGCCACTGAGAAG-3' | 462 | 1217472-1217933 |  |
|  |  | 5'-GTGCTTGTGACATGCGTAAGC-3' |  |  |  |

**Results：**

A total of 23 haplotypes were identified amongst 74 DNA sequences in *pvcsp* gene of 45 vivax malaria recurrent cases samples. The number of Variable sites and Expected heterozygosity (He) were 126, 0.9616, respectively. A total of 28 haplotypes were identified amongst 85 DNA sequences in *pvmsp-1* gene of 45 vivax malaria recurrent cases samples. The number of Variable sites and Expected heterozygosity (He) were 199, 0.9536, respectively. The results of gene sequence alignment of every vivax malaria recurrent cases were as follows:

1. Case 1

1.1 The alignment of *csp* partial gene （Variable sites=0, He=0, Haplotypes=1）


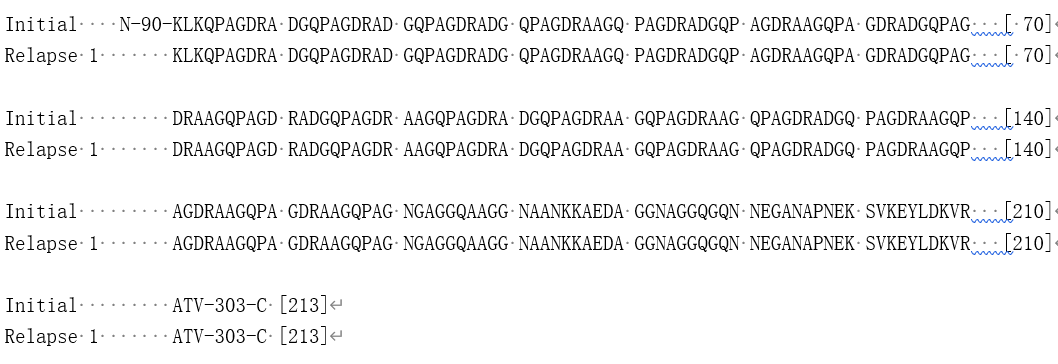


1.2 The alignment of *msp-1* partial gene （Variable sites=0, He=0, Haplotypes=1）


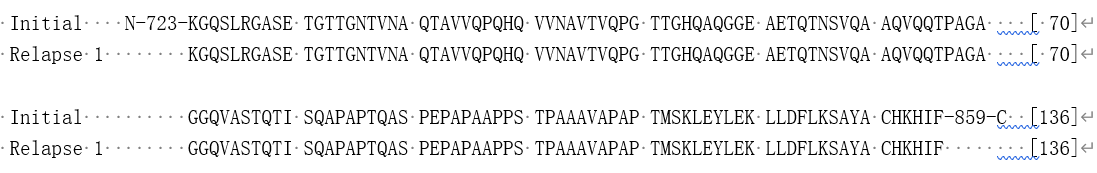


2. Case 2

2.1 The alignment of *csp* partial gene （Variable sites=0, He=0, Haplotypes=1）


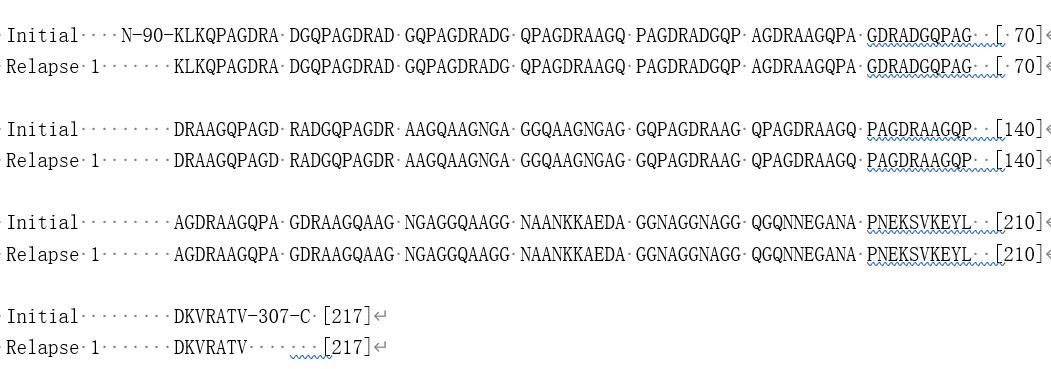


2.2 The alignment of *msp-1* partial gene （Variable sites=0, He=0, Haplotypes=1）


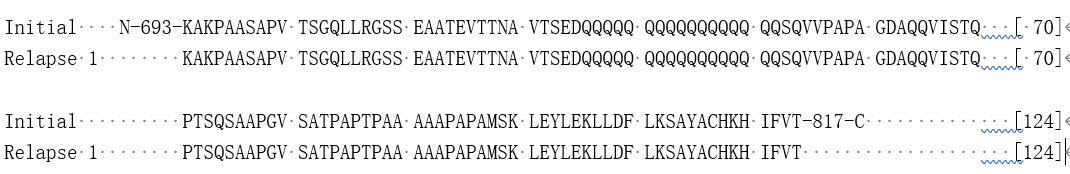


3. Case 3

3.1 The alignment of *csp* partial gene （Variable sites=0, He=0, Haplotypes=1）


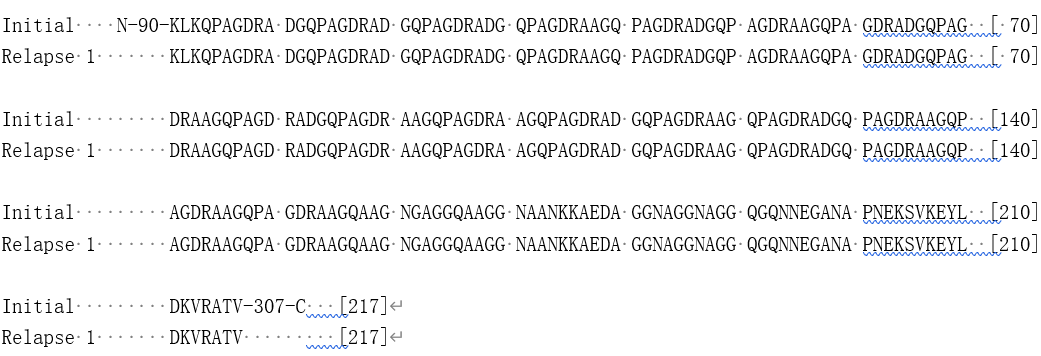


3.2 The alignment of *msp-1* partial gene （Variable sites=0, He=0, Haplotypes=1）


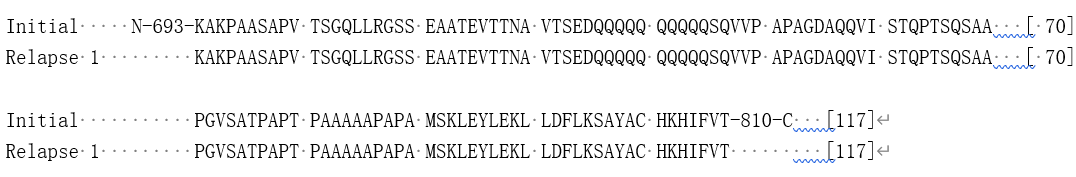


1. Case 4

4.1 The alignment of *csp* partial gene （Variable sites=0, He=0, Haplotypes=1）


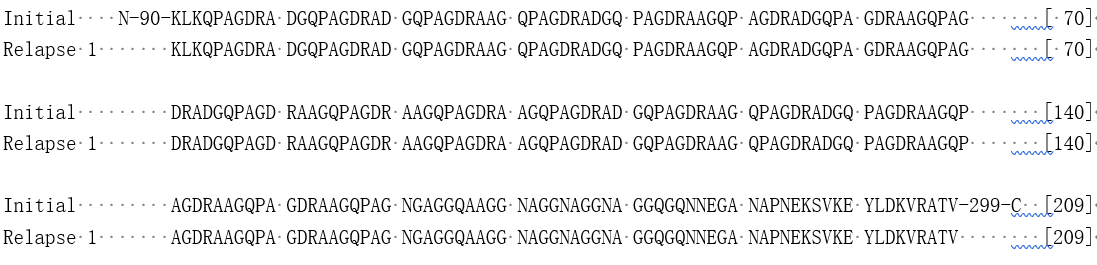


4.2 The alignment of *msp-1* partial gene （Variable sites=0, He=0, Haplotypes=1）


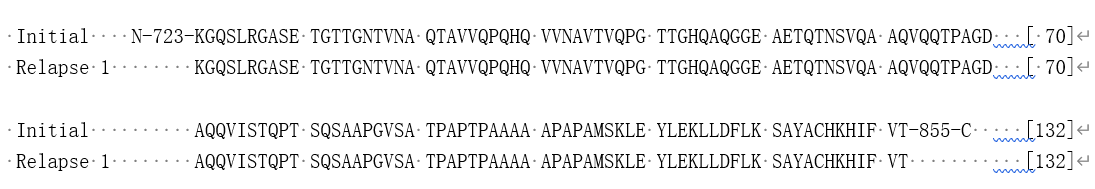


5. Case 5

5.1 The alignment of *csp* partial gene （Variable sites=0, He=0, Haplotypes=1）


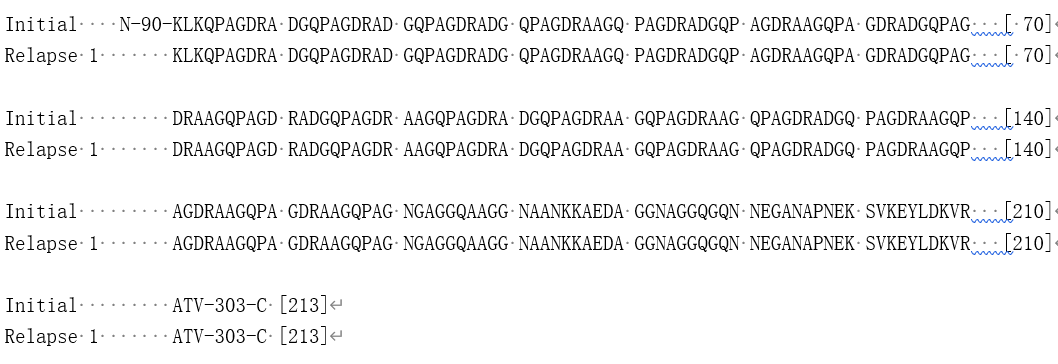


5.2 The alignment of *msp-1* partial gene （Variable sites=0, He=0, Haplotypes=1）


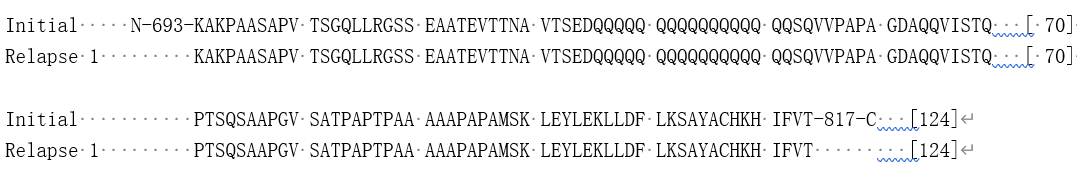


6. Case 6

6.1 The alignment of *csp* partial gene （Variable sites=0, He=0, Haplotypes=1）


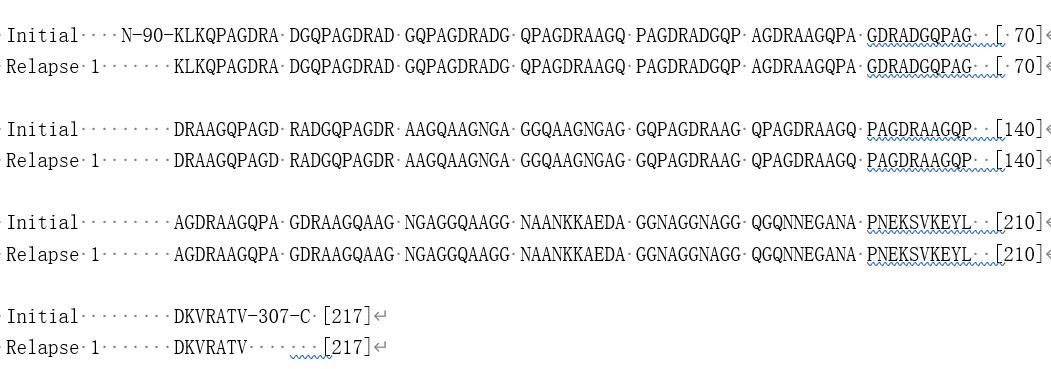


6.2 The alignment of *msp-1* partial gene （Variable sites=0, He=0, Haplotypes=1）


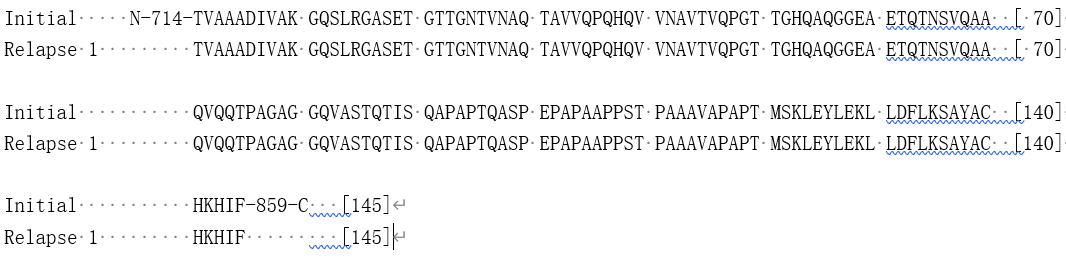


1. Case 7

7.1 The alignment of *csp* partial gene （Variable sites=0, He=0, Haplotypes=1）


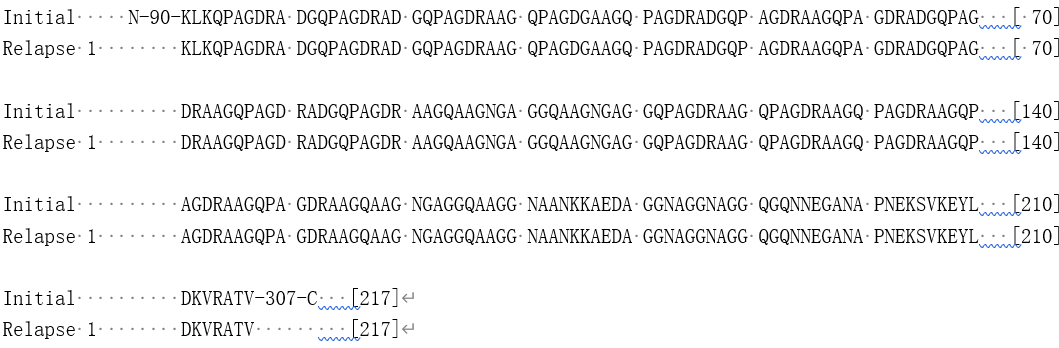


7.2 The alignment of *msp-1* partial gene （Variable sites=0, He=0, Haplotypes=1）


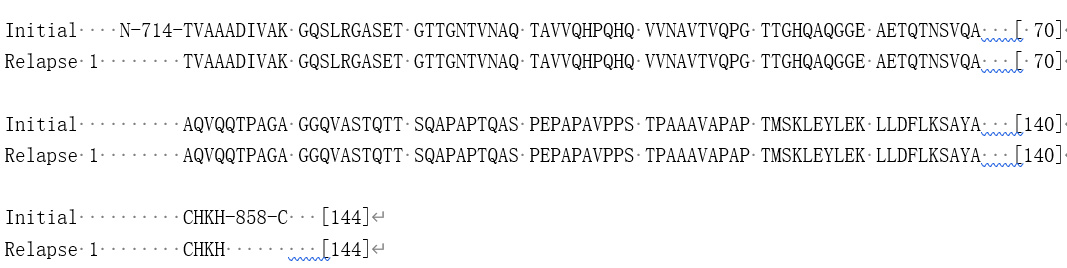


8. Case 8

8.1 The alignment of *csp* partial gene （Variable sites=0, He=0, Haplotypes=1）


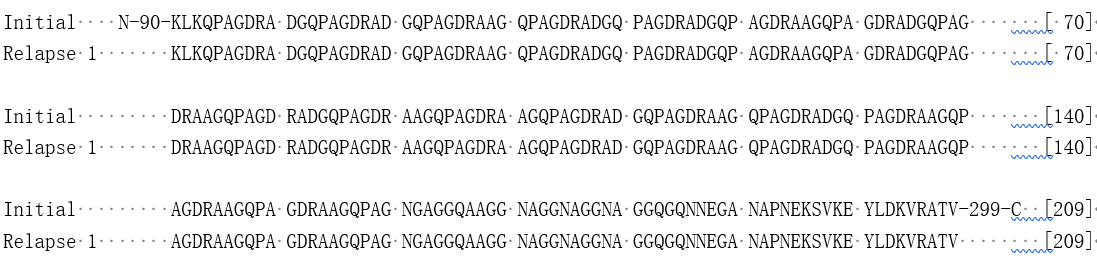


8.2 The alignment of *msp-1* partial gene （Variable sites=0, He=0, Haplotypes=1）


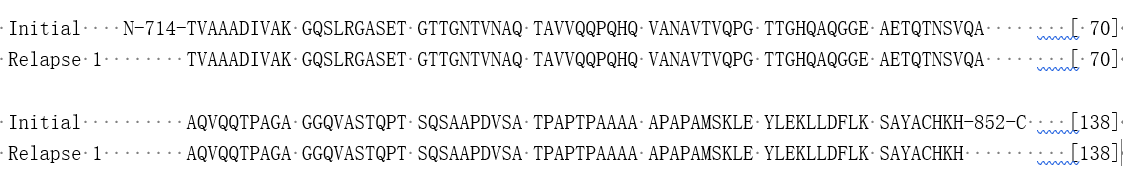


9. Case 9

9.1 The alignment of *csp* partial gene （Variable sites=0, He=0, Haplotypes=1）


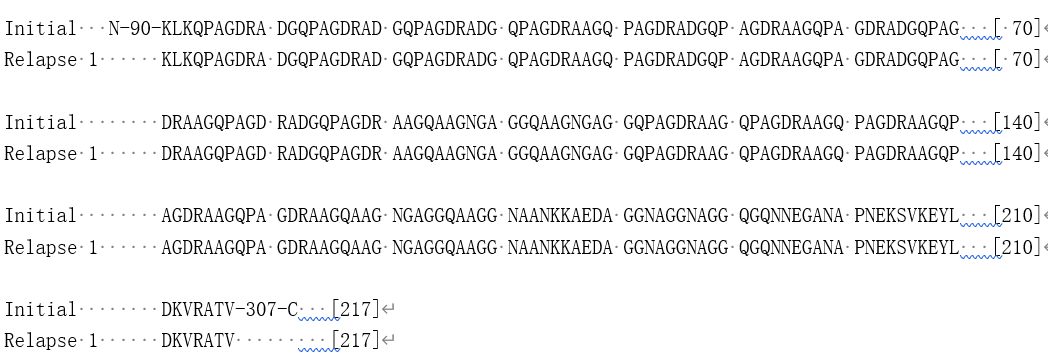


9.2 The alignment of *msp-1* partial gene （Variable sites=0, He=0, Haplotypes=1）


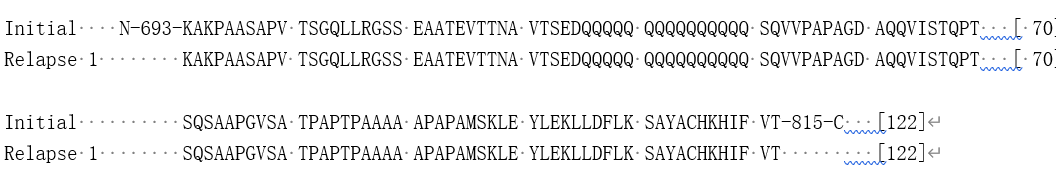


10. Case 10

10.1 The alignment of *csp* partial gene （Variable sites=0, He=0, Haplotypes=1）


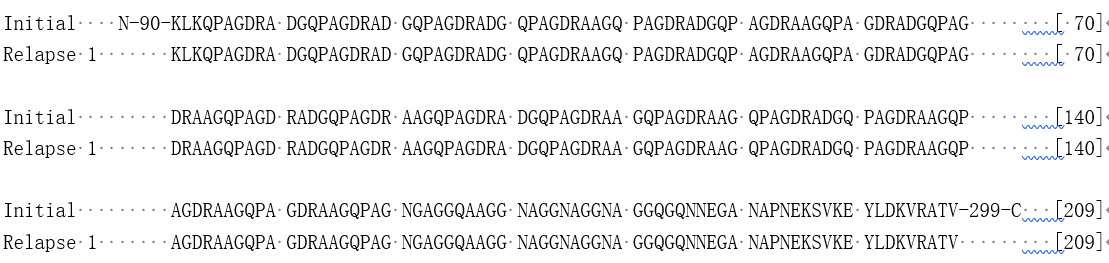


10.2 The alignment of *msp-1* partial gene （Variable sites=0, He=0, Haplotypes=1）


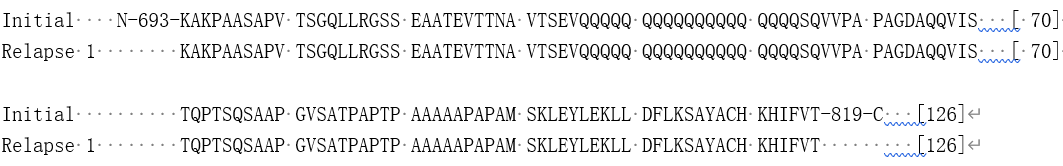


11. Case 11

11.1 The alignment of *csp* partial gene （Variable sites=0, He=0, Haplotypes=1）


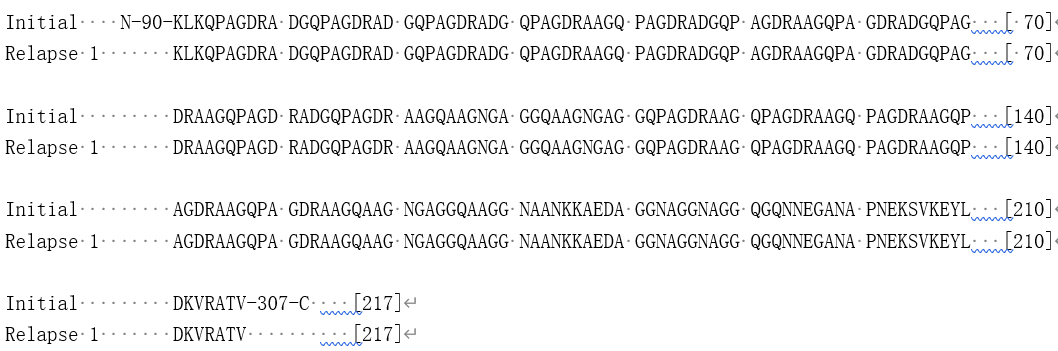


11.2 The alignment of *msp-1* partial gene （Variable sites=0, He=0, Haplotypes=1）


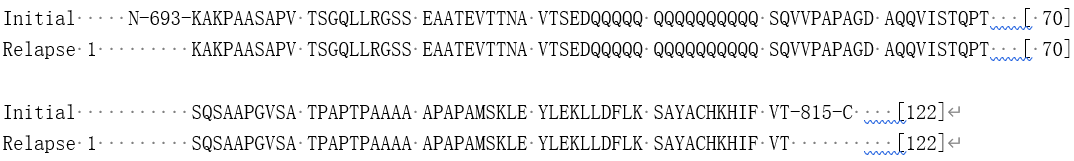


12. Case 12

12.1 The alignment of *csp* partial gene （Variable sites=0, He=0, Haplotypes=1）


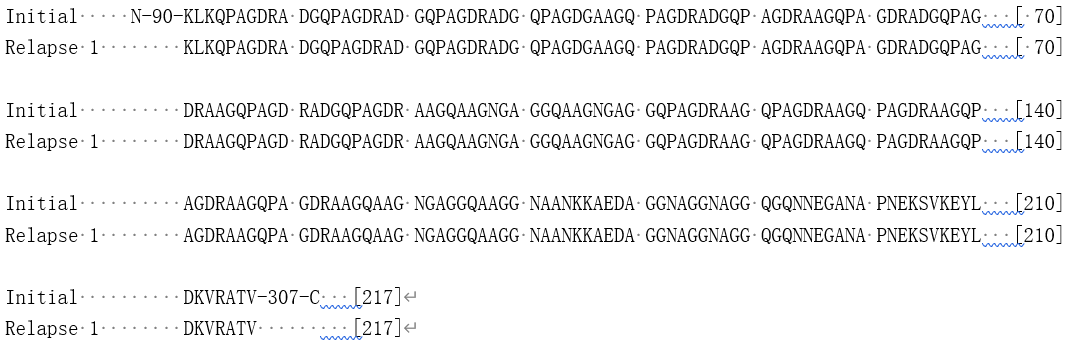


12.2 The alignment of *msp-1* partial gene （Variable sites=0, He=0, Haplotypes=1）


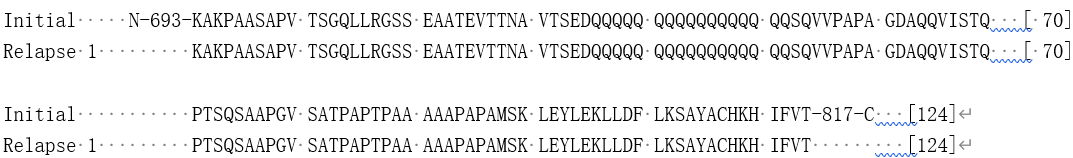


13. Case 13

13.1 The alignment of *csp* partial gene （Variable sites=0, He=0, Haplotypes=1）


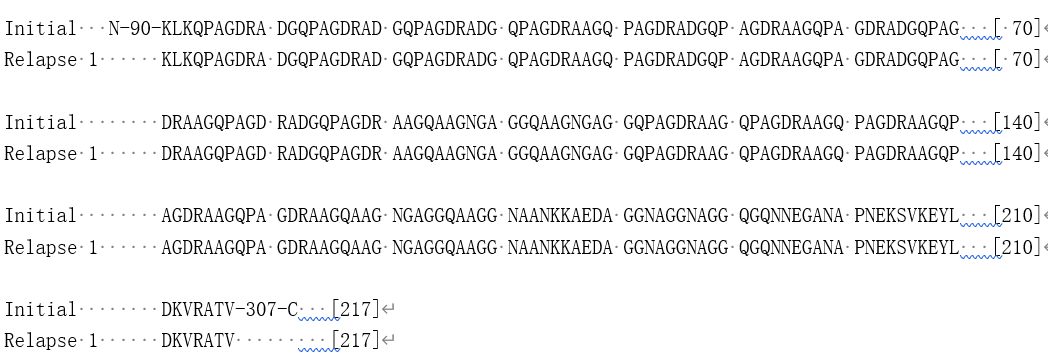


13.2 The alignment of *msp-1* partial gene （Variable sites=0, He=0, Haplotypes=1）


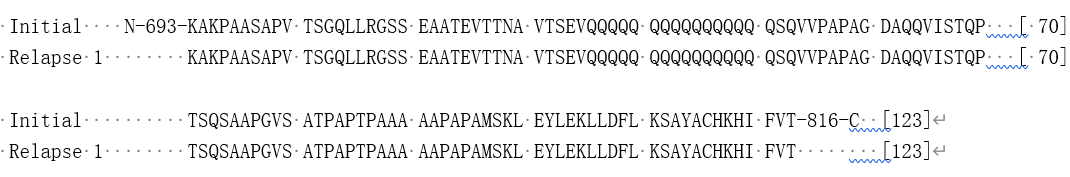


14. Case 14

14.1 The alignment of *csp* partial gene （Variable sites=0, He=0, Haplotypes=1）


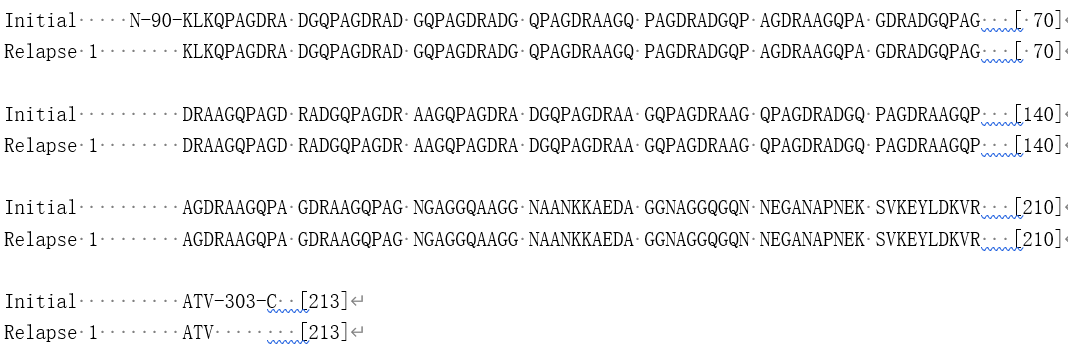


14.2 The alignment of *msp-1* partial gene （Variable sites=0, He=0, Haplotypes=1）


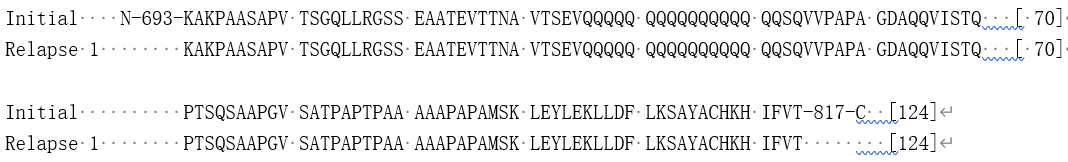


15. Case 15

15.1 The alignment of *csp* partial gene （Variable sites=0, He=0, Haplotypes=1）


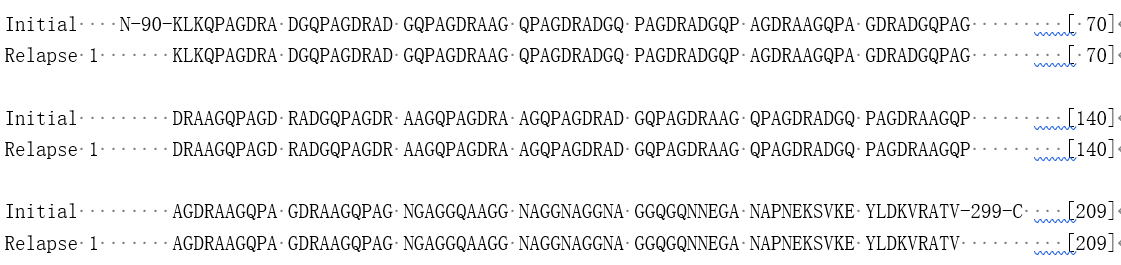


15.2 The alignment of *msp-1* partial gene （Variable sites=0, He=0, Haplotypes=1）


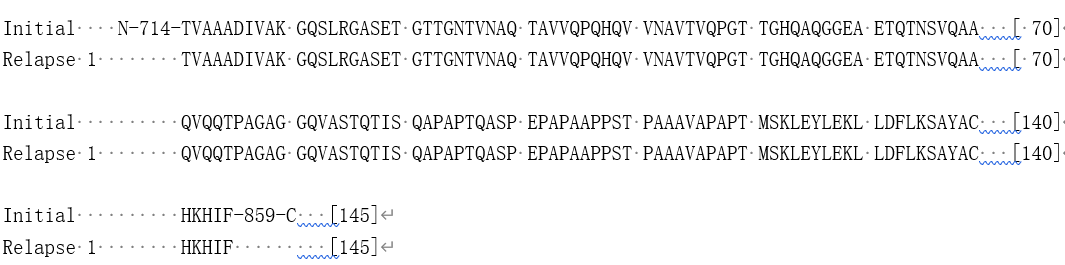


16. Case 16

16.1 The alignment of *csp* partial gene （Variable sites=0, He=0, Haplotypes=1）


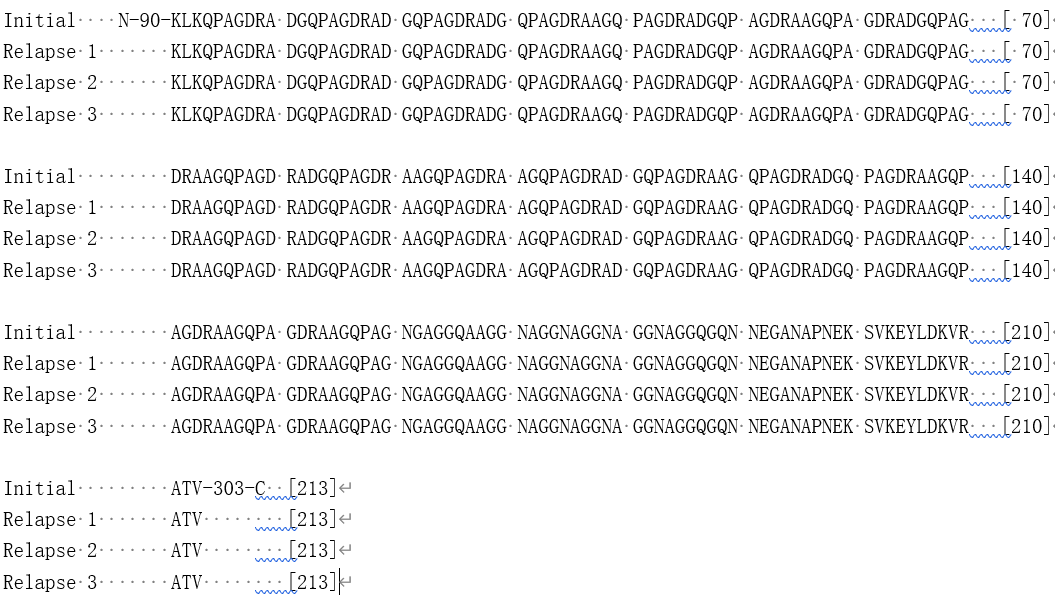


16.2 The alignment of *msp-1* partial gene （Variable sites=0, He=0, Haplotypes=1）


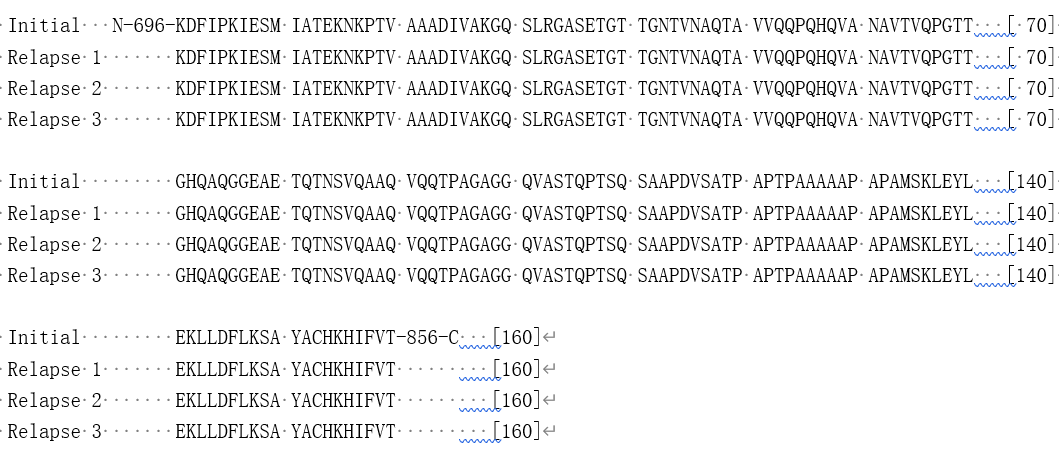


17. Case 17

17.1 The alignment of *csp* partial gene （Variable sites=0, He=0, Haplotypes=1）


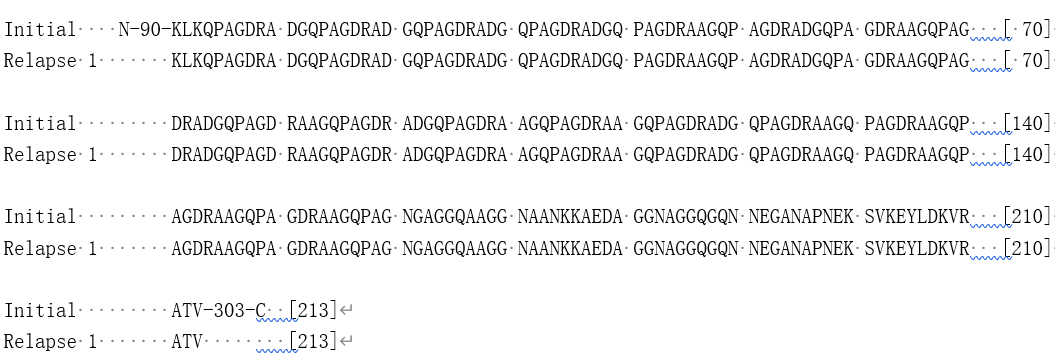


17.2 The alignment of *msp-1* partial gene （Variable sites=0, He=0, Haplotypes=1）


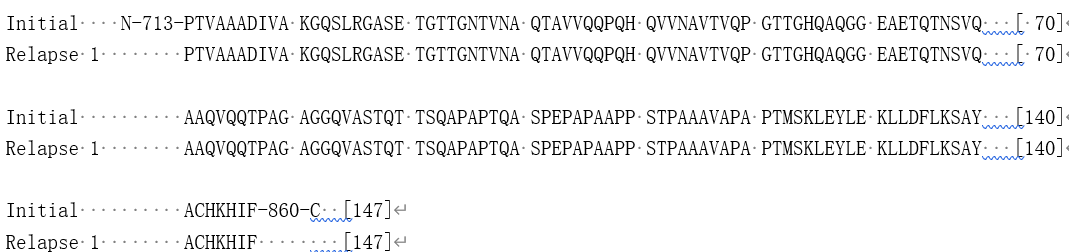


18. Case 18

18.1 The alignment of *csp* partial gene （Variable sites=0, He=0, Haplotypes=1）


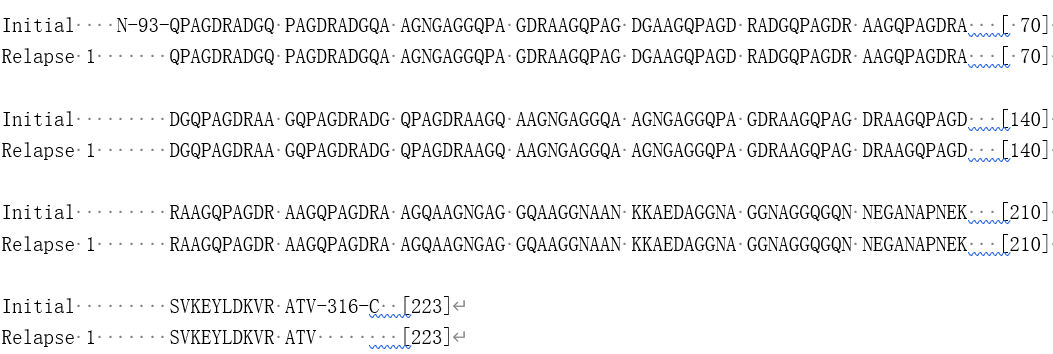


18.2 The alignment of *msp-1* partial gene （Variable sites=0, He=0, Haplotypes=1）


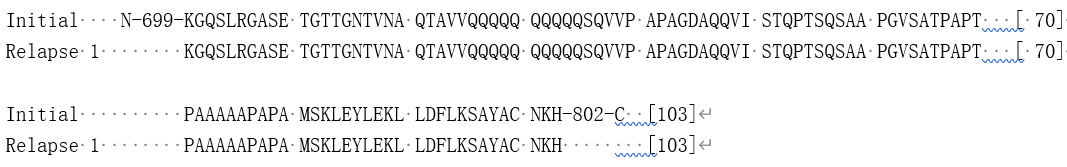


19. Case 19

19.1 The alignment of *csp* partial gene （Variable sites=0, He=0, Haplotypes=1）


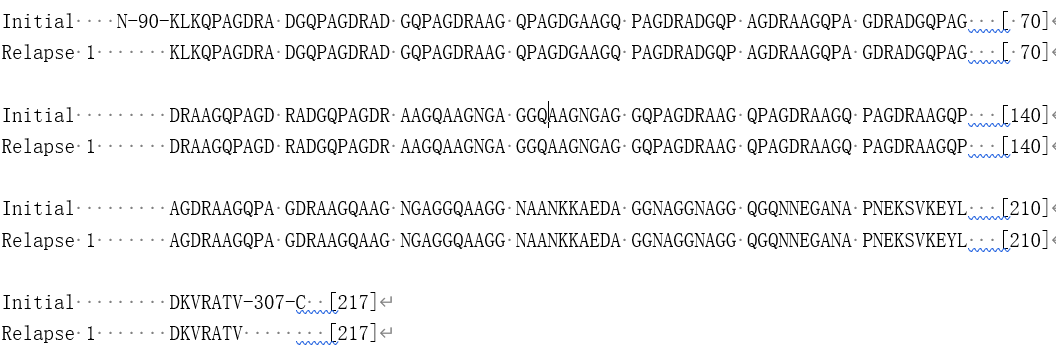


19.2 The alignment of *msp-1* partial gene （Variable sites=0, He=0, Haplotypes=1）


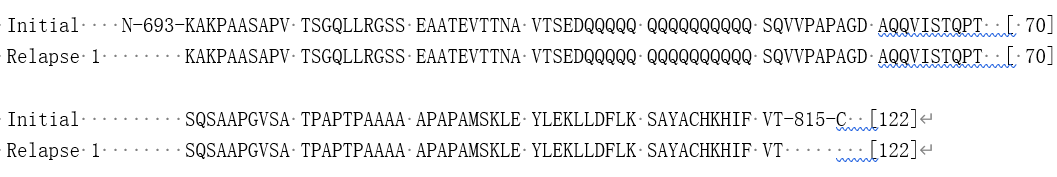


20. Case 20

20.1 The alignment of *csp* partial gene （Variable sites=0, He=0, Haplotypes=1）


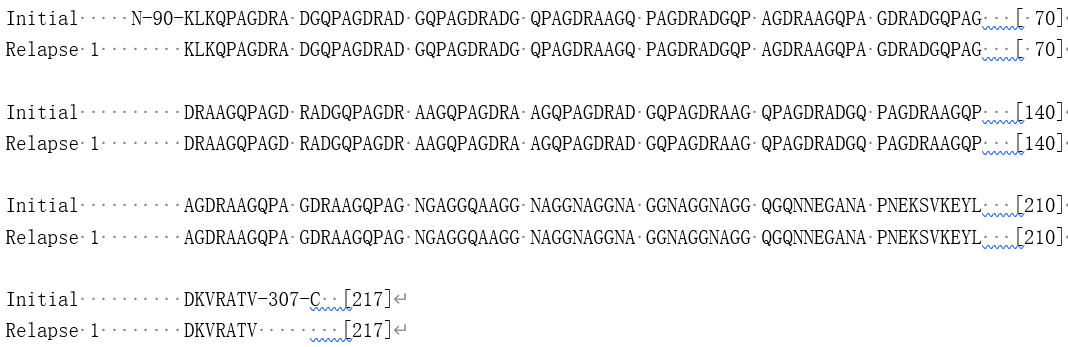


20.2 The alignment of *msp-1* partial gene （Variable sites=0, He=0, Haplotypes=1）


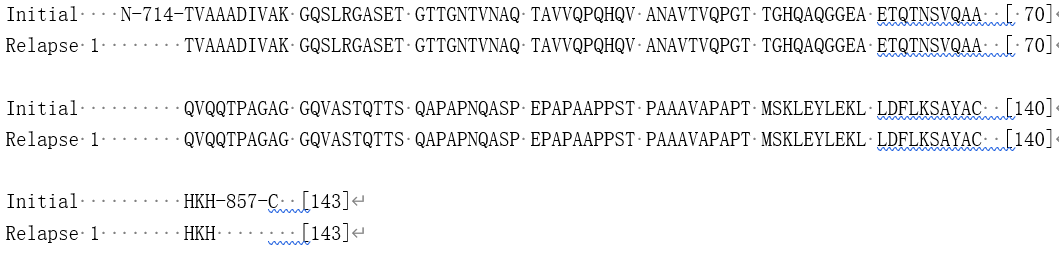


21. Case 21

21.1 The alignment of *csp* partial gene （Variable sites=0, He=0, Haplotypes=1）


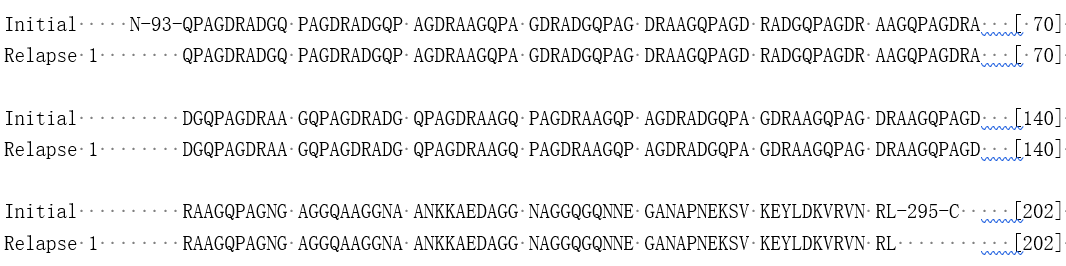


21.2 The alignment of *msp-1* partial gene （Variable sites=0, He=0, Haplotypes=1）


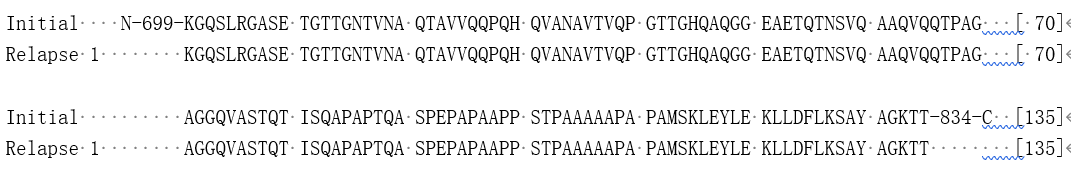


22. Case 22

22.1 The alignment of *csp* partial gene （Variable sites=0, He=0, Haplotypes=1）


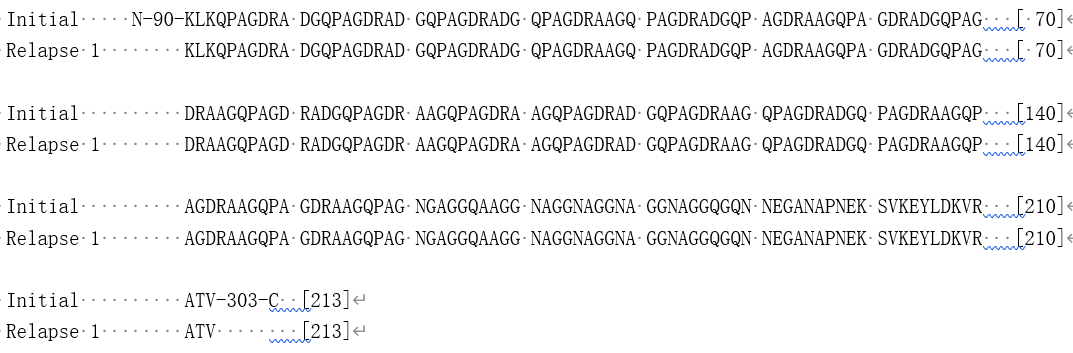


22.2 The alignment of *msp-1* partial gene （Variable sites=0, He=0, Haplotypes=1）


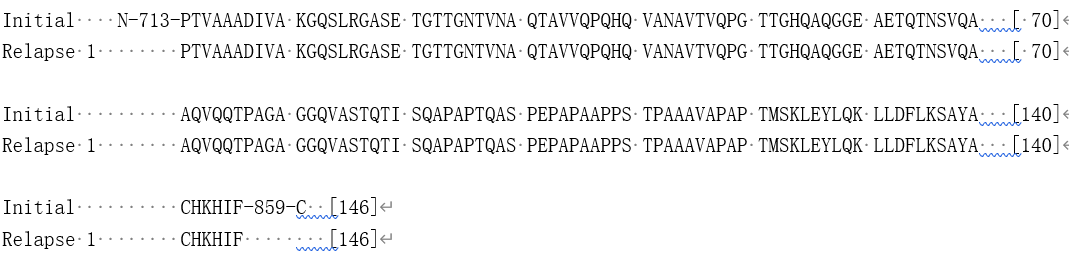


23. Case 23

The alignment of *msp-1* partial gene （Variable sites=0, He=0, Haplotypes=1）


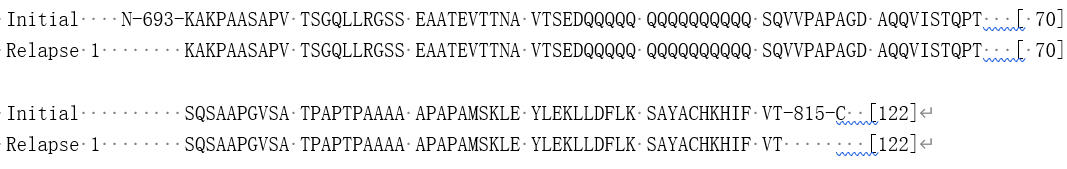


24. Case 24

24.1 The alignment of *csp* partial gene （Variable sites=0, He=0, Haplotypes=1）


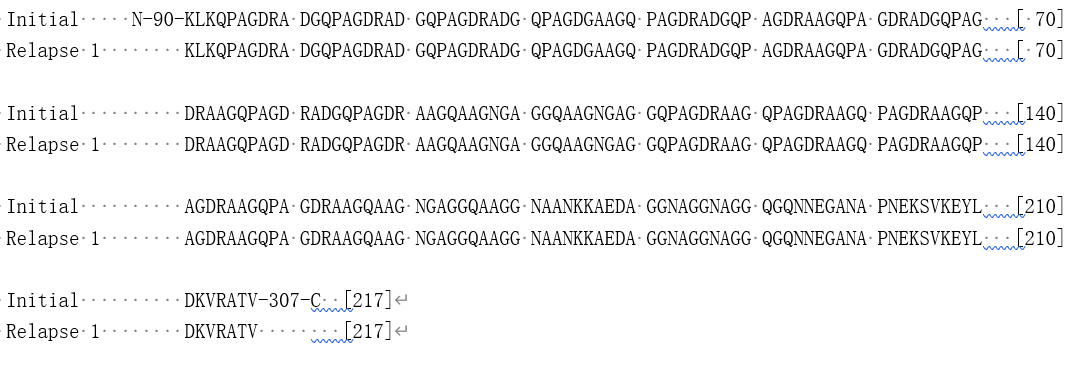


24.2 The alignment of *msp-1* partial gene （Variable sites=0, He=0, Haplotypes=1）


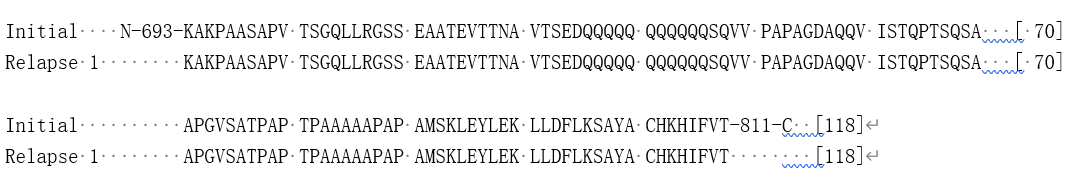


25. Case 25

The alignment of *msp-1* partial gene （Variable sites=0, He=0, Haplotypes=1）


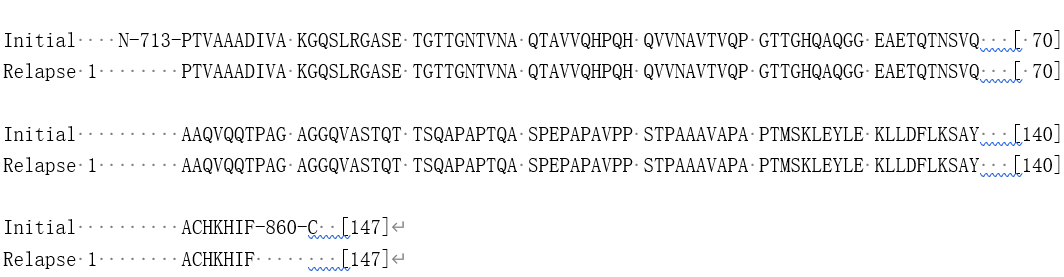


26. Case 26

The alignment of *csp* partial gene （Variable sites=0, He=0, Haplotypes=1）


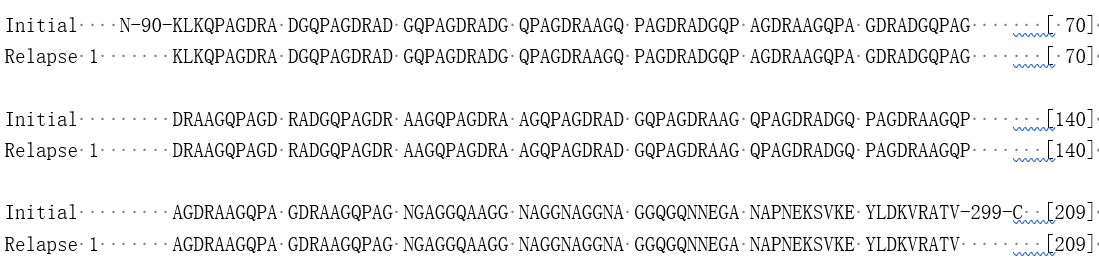


27. Case 27

27.1 The alignment of *csp* partial gene （Variable sites=0, He=0, Haplotypes=1）


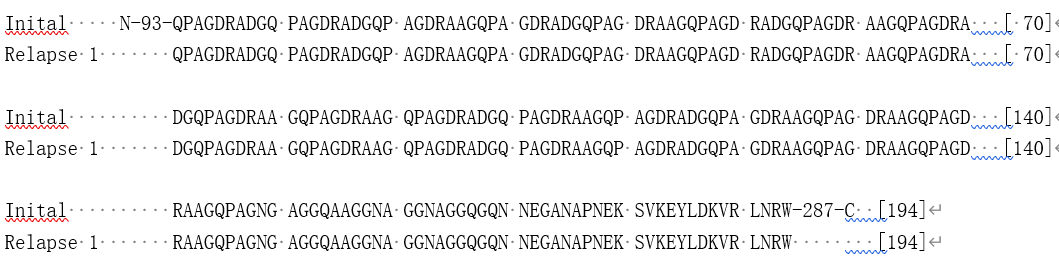


27.2 The alignment of *msp-1* partial gene （Variable sites=0, He=0, Haplotypes=1）


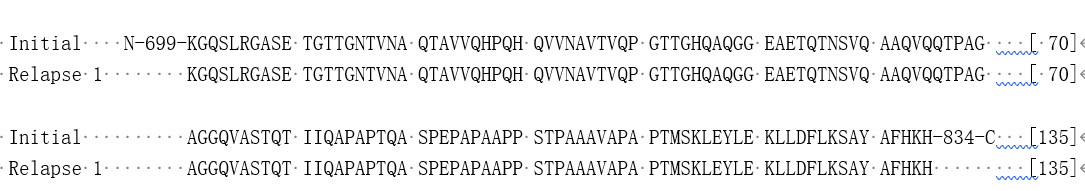


28. Case 28

28.1 The alignment of *csp* partial gene （Variable sites=0, He=0, Haplotypes=1）


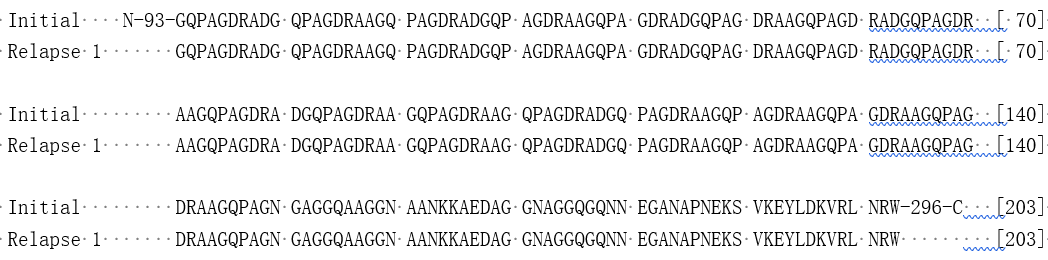


28.2 The alignment of *msp-1* partial gene （Variable sites=0, He=0, Haplotypes=1）


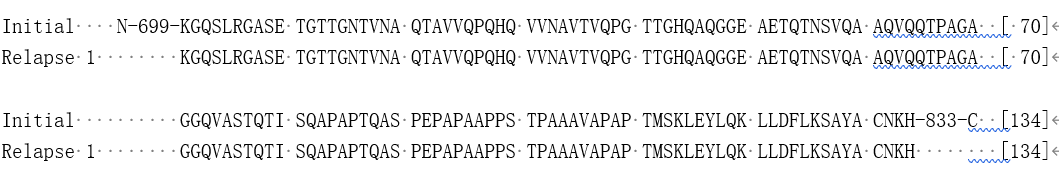


29. Case 29

The alignment of *msp-1* partial gene （Variable sites=0, He=0, Haplotypes=1）


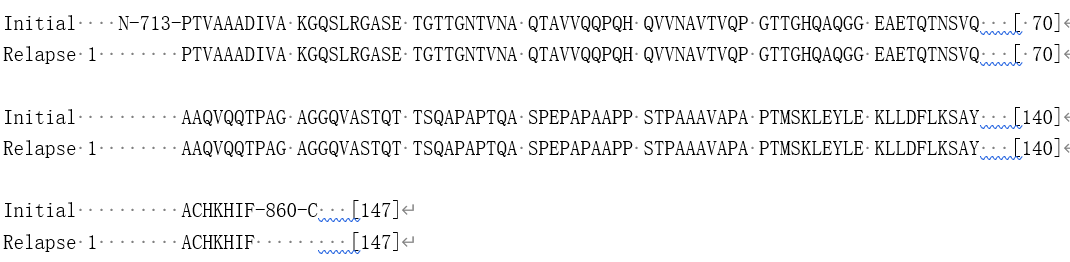


30. Case 30

The alignment of *msp-1* partial gene （Variable sites=0, He=0, Haplotypes=1）


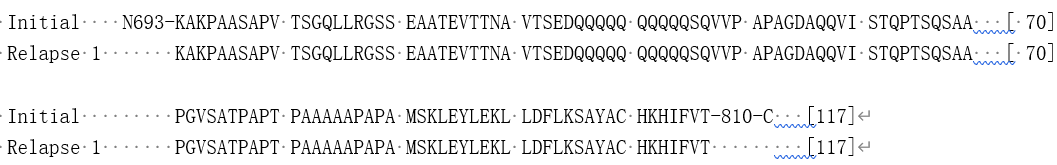


31. Case 31

The alignment of *msp-1* partial gene （Variable sites=0, He=0, Haplotypes=1）


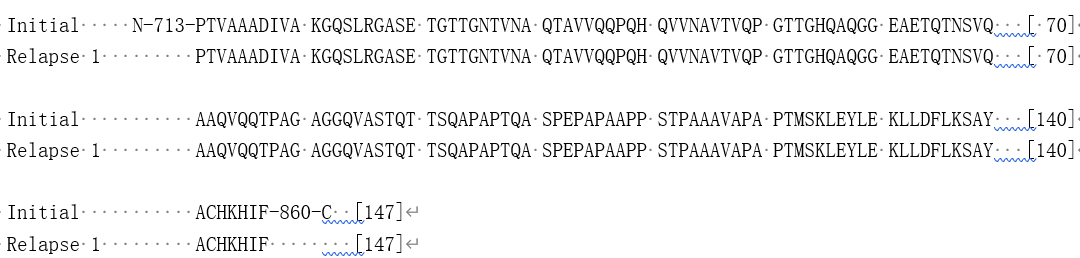


32. Case 32

32.1 The alignment of *csp* partial gene （Variable sites=0, He=0, Haplotypes=1）


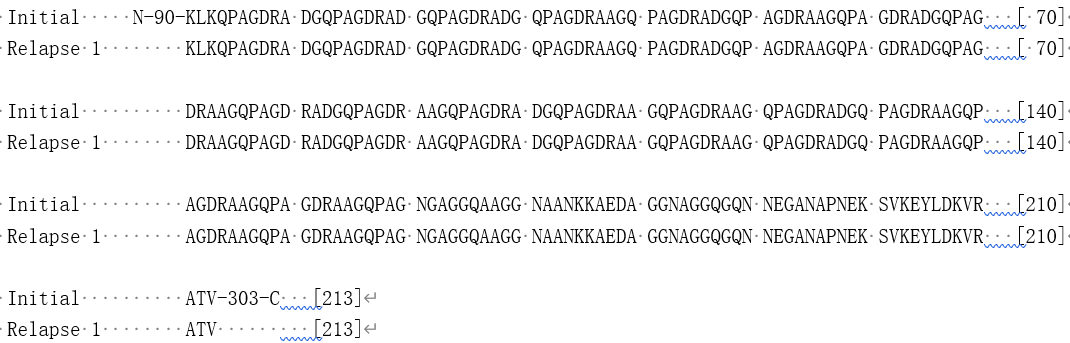


32.2 The alignment of *msp-1* partial gene （Variable sites=0, He=0, Haplotypes=1）


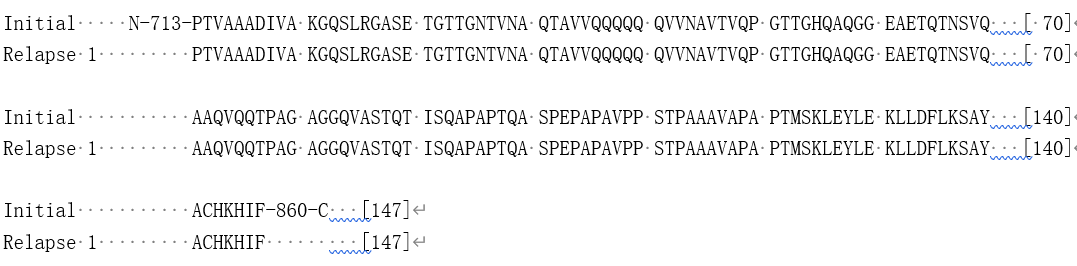


33. Case 33

The alignment of *csp* partial gene （Variable sites=0, He=0, Haplotypes=1）


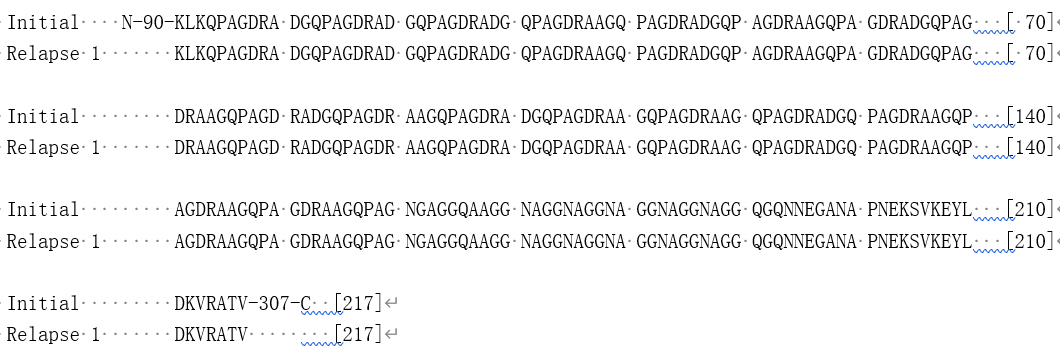


34. Case 34

34.1 The alignment of *csp* partial gene （Variable sites=0, He=0, Haplotypes=1）


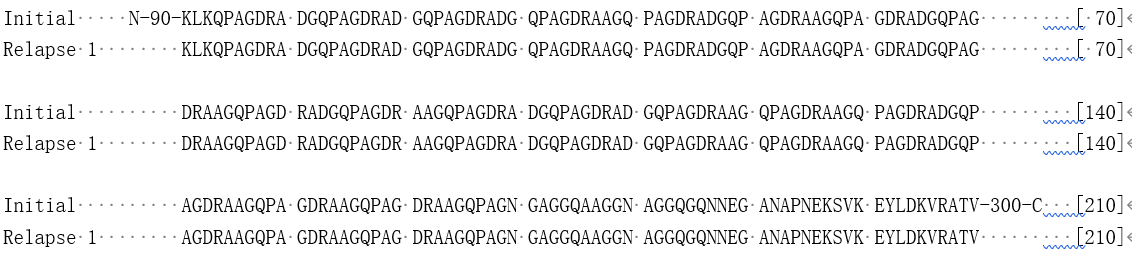


34.2 The alignment of *msp-1* partial gene （Variable sites=0, He=0, Haplotypes=1）


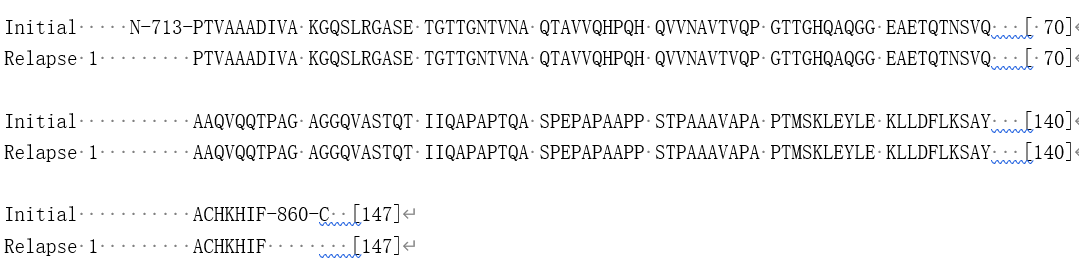


35. Case 35

The alignment of *msp-1* partial gene （Variable sites=0, He=0, Haplotypes=1）


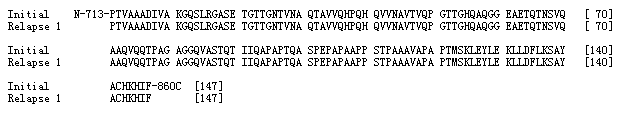


36. Case 36

The alignment of *msp-1* partial gene （Variable sites=0, He=0, Haplotypes=1）


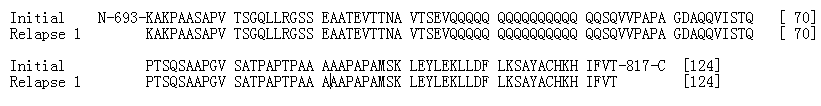


37. Case 37

37.1 The alignment of *csp* partial gene （Variable sites=0, He=0, Haplotypes=1）


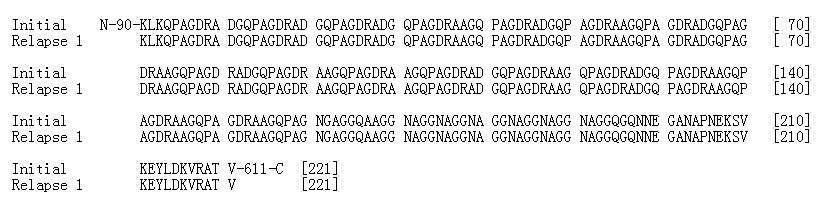


37.2 The alignment of *msp-1* partial gene （Variable sites=0, He=0, Haplotypes=1）


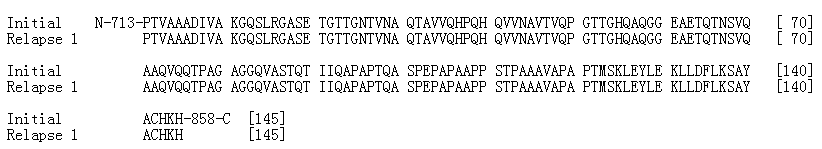


38. Case 38

38.1 The alignment of *csp* partial gene （Variable sites=0, He=0, Haplotypes=1）


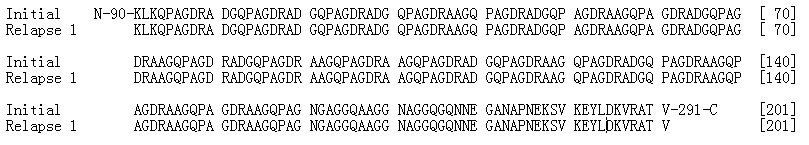


38.2 The alignment of *msp-1* partial gene （Variable sites=0, He=0, Haplotypes=1）


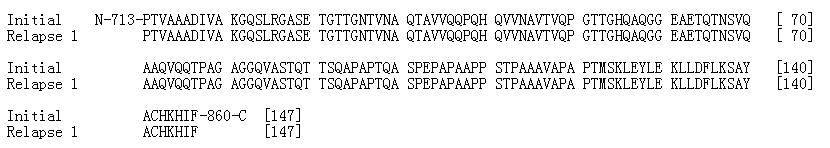


39. Case 39

39.1 The alignment of *csp* partial gene （Variable sites=0, He=0, Haplotypes=1）


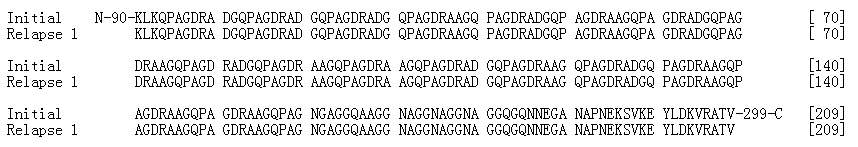


39.2 The alignment of *msp-1* partial gene （Variable sites=0, He=0, Haplotypes=1）


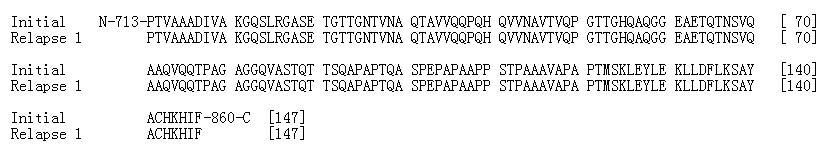


40. Case 40

40.1 The alignment of *csp* partial gene （Variable sites=0, He=0, Haplotypes=1）


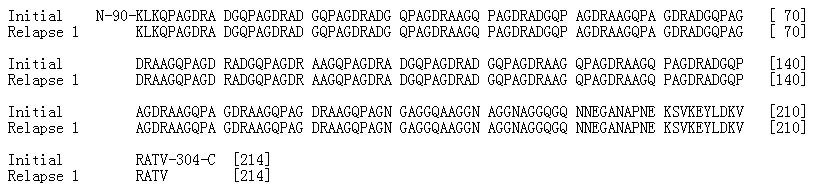


40.2 The alignment of *msp-1* partial gene （Variable sites=0, He=0, Haplotypes=1）


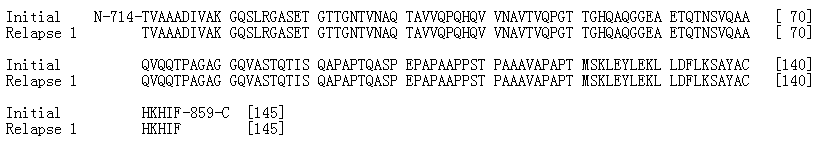


41. Case 41

41.1 The alignment of *csp* partial gene （Variable sites=0, He=0, Haplotypes=1）


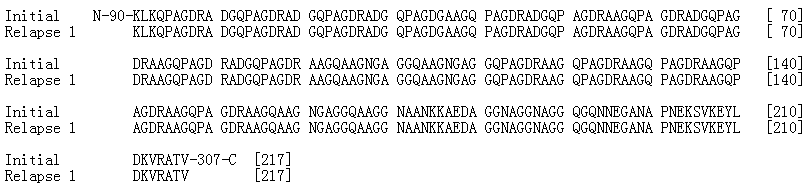


41.2 The alignment of *msp-1* partial gene （Variable sites=0, He=0, Haplotypes=1）


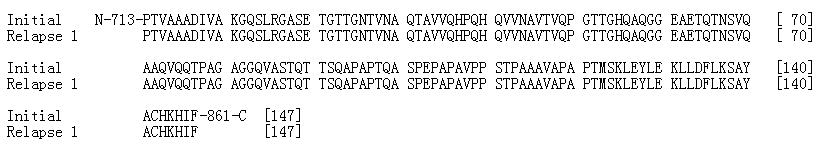


42. Case 42

42.1 The alignment of *csp* partial gene （Variable sites=0, He=0, Haplotypes=1）


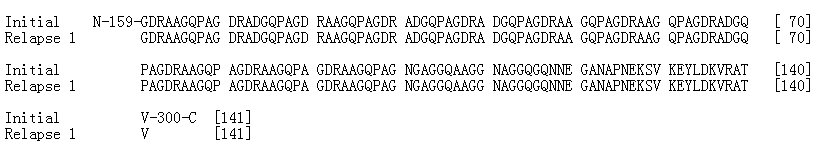


42.2 The alignment of *msp-1* partial gene （Variable sites=0, He=0, Haplotypes=1）


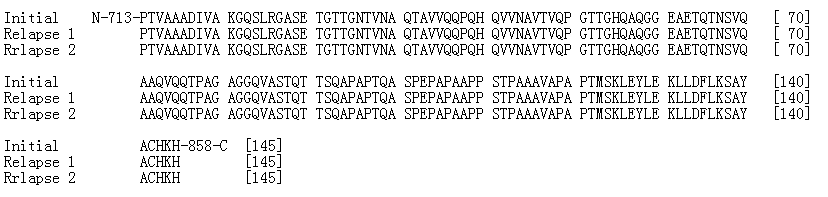


43. Case 43

The alignment of *msp-1* partial gene （Variable sites=0, He=0, Haplotypes=1）


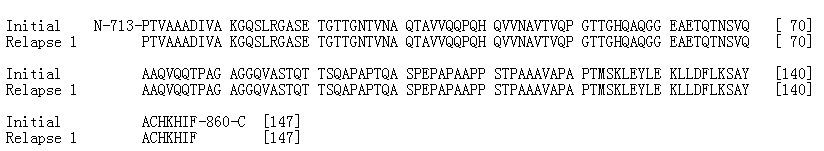


44. Case 44

The alignment of *msp-1* partial gene （Variable sites=0, He=0, Haplotypes=1）


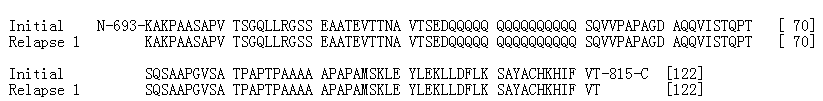


45. Case 45

45.1 The alignment of *csp* partial gene （Variable sites=0, He=0, Haplotypes=1）


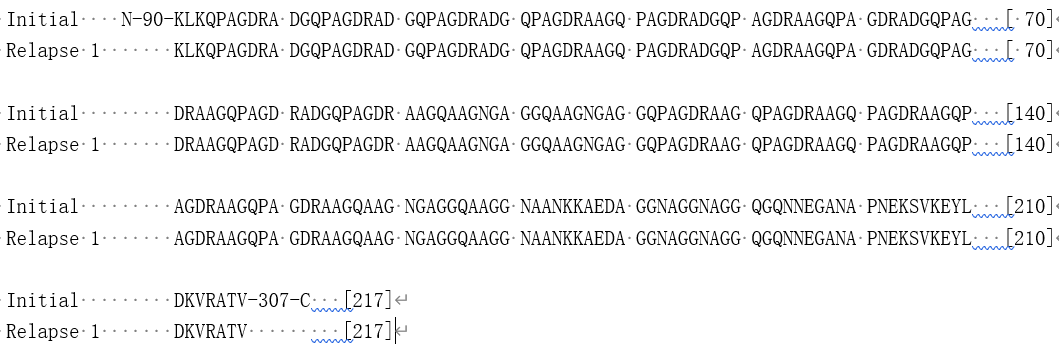


45.2 The alignment of *msp-1* partial gene （Variable sites=0, He=0, Haplotypes=1）


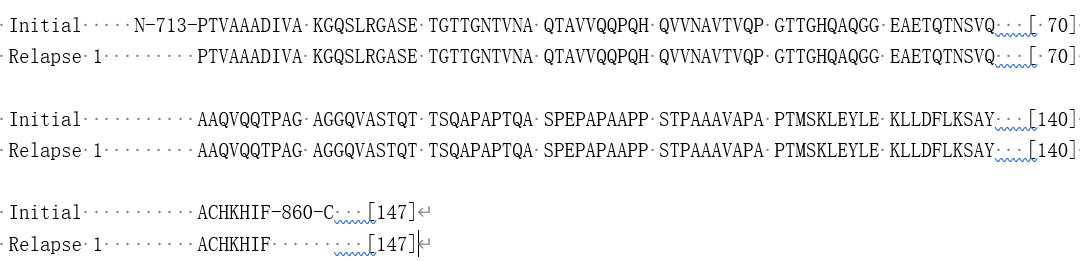

Supplement: Supplementary file 3 — Additional file 3. Epidemiological information and alignment results of the genes of suspected recurrent cases of vivax malaria. [file 12936_2021_3685_MOESM3_ESM.doc]
