# Supplementary material for: The association of CYP2D6 gene polymorphisms in the full-length coding region with higher recurrence rate of vivax malaria in Yunnan Province, China
Source: Malar J. 2021 Mar 20;20:160. doi: 10.1186/s12936-021-03685-3 (PMC7981985; doi:10.1186/s12936-021-03685-3)
Supplement: Supplementary file 4 — Additional file 4. Electrophoretic map of PCR products in the coding region of CYP2D6 gene. [file 12936_2021_3685_MOESM4_ESM.docx]

**Additional file 4**

PCR products of CYP2D6 gene fragment


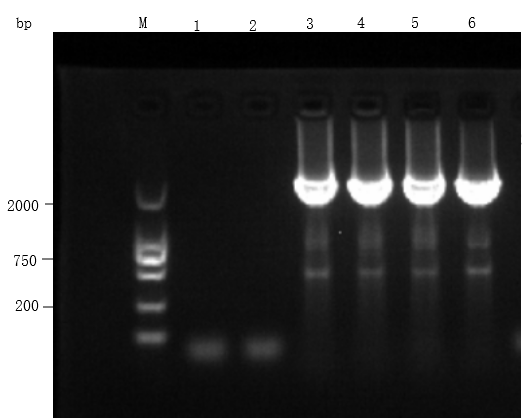

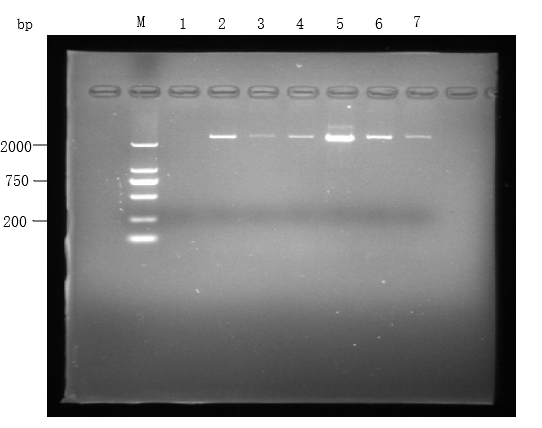


a b

**Fig. S4 a** M: DNA marker; 1: blank control of PCR for the first round; 2: blank control of PCR for the second round; 3-6: The amplification products of exon 1-4 in CYP2D6 gene; **b** M: DNA marker; 1: blank control of PCR; 2-7: The amplification products of exon 5-9 in CYP2D6 gene.
