# Supplementary material for: The association of CYP2D6 gene polymorphisms in the full-length coding region with higher recurrence rate of vivax malaria in Yunnan Province, China
Source: Malar J. 2021 Mar 20;20:160. doi: 10.1186/s12936-021-03685-3 (PMC7981985; doi:10.1186/s12936-021-03685-3)
Supplement: Supplementary file 5 — Additional file 5. Analysis the CYP2D6 genotypes in SR group and NR group. [file 12936_2021_3685_MOESM5_ESM.docx]

| **Additional file 5 Analysis the genotypes in SR group and NR group** | | | | | | | | | | | | | | | | |
| --- | --- | --- | --- | --- | --- | --- | --- | --- | --- | --- | --- | --- | --- | --- | --- | --- |
| **Genotypes** | **Suballele**  **genotypes** | **Mutation loci and allelic form^a^** | | | | | | | | | | | |  | **Case groups** | |
|  |  | **c.31** | **c.100** | **c.271** | **c.281** | **c.294** | **c.297** | **c.336** | **c.408** | **c.505** | **c.801** | **c.886** | **c.1457** |  | **SR group**  **No. (n=44, F/%)** | **NR group No. (n=75, F/%)** |
| **NC_000022.11** | -- | G/G | C/C | C/C | A/A | C/C | C/C | C/C | G/G | G/G | C/C | C/C | G/G |  | -- | -- |
| ***1/*1** | *1.001/*1.001 | －/－ | －/－ | －/－ | －/－ | －/－ | －/－ | －/－ | －/－ | －/－ | －/－ | －/－ | －/－ |  | 1(2.3) | 11(14.7) |
| ***2/*2** | *2.025/*2.025 | －/－ | －/－ | －/－ | －/－ | －/－ | －/－ | －/－ | ***C/C*** | －/－ | ***A/A*** | ***T/T*** | ***C/C*** |  | 4(9.1) | 1(1.3) |
|  | *2.001/*2.001 | －/－ | －/－ | －/－ | －/－ | －/－ | －/－ | －/－ | ***C/C*** | －/－ | －/－ | ***T/T*** | ***C/C*** |  |  |  |
| ***4/*4** | *4.001/*4.001 | －/－ | ***T/T*** | ***A/A*** | ***G/G*** | ***G/G*** | －/－ | －/－ | ***C/C*** | －/－ | －/－ | －/－ | ***C/C*** |  | 1(2.3) | 0 |
| ***4/*o** | *4.001/*o | －/－ | ***T/T*** | ***A/A*** | ***G/G*** | ***G/G*** | －/T | －/－ | ***C/C*** | －/－ | －/－ | －/－ | ***C/C*** |  | 1(2.3) | 0 |
| ***1/*2** | *1.001/*2.004 | －/－ | －/－ | －/－ | －/－ | －/－ | －/－ | －/－ | －/－ | －/－ | －/－ | －/T | －/C |  | 4(9.1) | 1(1.3) |
|  | *1.001/*2.001 | －/－ | －/－ | －/－ | －/－ | －/－ | －/－ | －/－ | －/C | －/－ | －/－ | －/T | －/C |  |  |  |
| ***2/*39** | *2.001/*39.001 | －/－ | －/－ | －/－ | －/－ | －/－ | －/－ | －/－ | ***C/C*** | －/－ | －/－ | －/T | ***C/C*** |  | 2(4.5) | 0 |
|  | *2.002/*39.001 | －/－ | －/－ | －/－ | －/－ | －/－ | －/－ | －/T | ***C/C*** | －/－ | －/－ | －/T | ***C/C*** |  |  |  |
| ***39/*t** | *39.001/*t | －/－ | －/T | －/－ | －/－ | －/－ | －/－ | －/－ | ***C/C*** | －/－ | －/－ | －/T | ***C/C*** |  | 0 | 1(1.3) |
| ***39/*m** | *39.001/*m | －/－ | －/－ | －/－ | －/－ | －/－ | －/－ | －/－ | ***C/C*** | －/A | －/－ | －/－ | ***C/C*** |  | 1(2.3) | 0 |
| ***39/*x** | *39.001/*x | －/－ | －/T | －/－ | －/－ | －/－ | －/－ | －/T | ***C/C*** | －/A | －/－ | －/T | ***C/C*** |  | 1(2.3) | 0 |
| ***39/*s** | *39.001/*s | －/－ | －/T | －/－ | －/－ | －/－ | －/－ | －/T | ***C/C*** | －/－ | －/－ | －/T | ***C/C*** |  | 2(4.5) | 7(9.4) |
| ***s/*s** | *s/*s | －/－ | ***T/T*** | －/－ | －/－ | －/－ | －/－ | ***T/T*** | ***C/C*** | －/－ | －/－ | ***T/T*** | ***C/C*** |  | 1(2.3) | 0 |
| ***10/*s** | *10.002/*s | －/－ | ***T/T*** | －/－ | －/－ | －/－ | －/－ | ***T/T*** | ***C/C*** | －/－ | －/－ | －/T | ***C/C*** |  | 1(2.3) | 1(1.3) |
| ***10/*s** | *10.001/*s | －/－ | ***T/T*** | －/－ | －/－ | －/－ | －/－ | －/T | ***C/C*** | －/－ | －/－ | －/T | ***C/C*** |  | 0 | 7(9.4) |
| ***1/*t** | *1.001/*t | －/－ | －/T | －/－ | －/－ | －/－ | －/－ | －/－ | －/C | －/－ | －/－ | －/T | －/C |  | 0 | 1(1.3) |
| ***1/*r** | *1.011/*r | －/－ | －/－ | －/－ | －/－ | －/－ | －/－ | －/－ | ***C/C*** | －/－ | －/－ | －/T | －/－ |  | 1(2.3) | 0 |
| ***1/*n** | *1.001/*n | －/A | －/T | －/－ | －/－ | －/－ | －/－ | －/T | －/C | －/－ | －/－ | －/T | －/C |  | 1(2.3) | 0 |
| ***1/*u** | *1.001/*u | －/－ | －/－ | －/－ | －/－ | －/－ | －/－ | －/T | －/－ | －/－ | －/－ | －/－ | －/－ |  | 0 | 1(1.3) |
| ***39/*39** | *39.001/*39.002 | －/－ | －/－ | －/－ | －/－ | －/－ | －/－ | －/－ | －/C | －/－ | －/－ | －/－ | ***C/C*** |  | 2(4.5) | 1(1.3) |
|  | *39.002/*39.002 | －/－ | －/－ | －/－ | －/－ | －/－ | －/－ | －/－ | －/－ | －/－ | －/－ | －/－ | ***C/C*** |  |  |  |
| ***10/*39** | *10.001/*39.001 | －/－ | －/T | －/－ | －/－ | －/－ | －/－ | －/－ | ***C/C*** | －/－ | －/－ | －/－ | ***C/C*** |  | 5(11.4) | 3(4.0) |
|  | *10.002/*39.001 | －/－ | －/T | －/－ | －/－ | －/－ | －/－ | －/T | ***C/C*** | －/－ | －/－ | －/－ | ***C/C*** |  |  |  |
| ***10/*10** | *10.002/*10.002 | －/－ | ***T/T*** | －/－ | －/－ | －/－ | －/－ | ***T/T*** | ***C/C*** | －/－ | －/－ | －/－ | ***C/C*** |  | 12(27.2) | 24(32.0) |
|  | *10.001/*10.002 | －/－ | ***T/T*** | －/－ | －/－ | －/－ | －/－ | －/T | ***C/C*** | －/－ | －/－ | －/－ | ***C/C*** |  |  |  |
| ***1/*10** | *1.001/*10.002 | －/－ | －/T | －/－ | －/－ | －/－ | －/－ | －/T | －/C | －/－ | －/－ | －/－ | －/C |  | 2(4.5) | 12(16.0) |
|  | *1.011/*10.001 | －/－ | －/T | －/－ | －/－ | －/－ | －/－ | －/－ | ***C/C*** | －/－ | －/－ | －/－ | －/C |  |  |  |
|  | *1.011/*10.002 | －/－ | －/T | －/－ | －/－ | －/－ | －/－ | －/T | ***C/C*** | －/－ | －/－ | －/－ | －/C |  |  |  |
| ***v/*10** | *v/*10.002 | －/－ | ***T/T*** | －/－ | －/－ | －/－ | －/－ | ***T/T*** | ***C/C*** | －/－ | －/－ | －/－ | －/C |  | 2(4.5) | 0 |
| ***w/*10** | *w/*10.002 | －/－ | ***T/T*** | －/－ | －/－ | －/－ | －/－ | －/T | ***C/C*** | －/－ | －/－ | －/－ | －/C |  | 0 | 3(4.0) |
| ***p/*q** | *p/*q | －/－ | －/T | ***T/T*** | －/－ | －/G | －/－ | ***T/T*** | －/C | －/－ | －/－ | －/－ | －/C |  | 0 | 1(1.3) |
| ***5/*5^b^** | -- | -- | -- | -- | -- | -- | -- | -- | -- | -- | -- | -- | -- |  | -- | -- |
| Note: ^a^ DNA base highlighted in bold indicates the occurrence of SNP; －: non-mutation; n: number of cases; ^b^Amplification failure, CYP2D6 full gene deletion; F: Frequency; SR: Suspected relapsed cases of vivax malaria; NR: Non-relapsed cases of vivax malaria; *m-*x: They could not meet the allele inclusion criteria provided by the Allele Nomenclature Committee[33]. | | | | | | | | | | | | | | | | |
